# Oxygen-induced multimodal ultramicroporous structure in 10-nm-thick carbon membranes for enhanced hydrogen separation

**DOI:** 10.1038/s41467-026-73556-5

**Published:** 2026-05-22

**Authors:** Yueqing Shen, Cédric Van Goethem, Heng-Yu Chi, Yinghui Li, Linyi Li, Nicole Barber, Kuang-Jung Hsu, Daniel Ortiz Trujillo, Natalia Gasilova, Adam Squires, Shiqi Huang, Kumar Varoon Agrawal

**Affiliations:** 1 Laboratory of Advanced Separations, École Polytechnique Fédérale de Lausanne (EPFL), Sion, Switzerland; 2 Laboratory of Materials for Renewable Energy, École Polytechnique Fédérale de Lausanne (EPFL), Sion, Switzerland; 3 Centre for Integrated Materials, Processes & Structures (IMPS), Department of Chemical Engineering, University of Bath, Bath, UK; 4 Department of Chemistry, University of Bath, Bath, UK; 5 Mass Spectrometry and Elemental Analysis Platform (MSEAP), École Polytechnique Fédérale de Lausanne (EPFL), Lausanne, Switzerland

**Keywords:** Synthesis and processing, Chemical engineering

## Abstract

Carbon membranes yielding high selectivity as well as high permeance are attractive to advance the membrane-based gas separation. Herein, we report ultrathin carbon membranes (UCMs) which deliver enhanced gas separation performance through oxygen-modulated pyrolysis of poly(4-vinylpyridine) precursor. We show that O_2_ in pyrolysis environment, transforms the otherwise uniform carbon network featuring a ~ 3.9 Å characteristic interlayer spacing into disrupted UCMs (d-UCMs). These d-UCMs possess a multimodal ultramicroporous structure characterized by distinct d-spacings of ~3.4 Å, 3.9 Å, and 5.5 Å. This optimized distribution of free volume in a 10-nm-thick membrane enables a record combination of H_2_ permeance exceeding 10,000 gas permeation units (GPUs) and H_2_/N_2_ mixture selectivity surpassing 200. Meanwhile, d-UCM exhibits physical and thermal stability, showing no aging over 7 days of elevated temperature permeance testing, which overcomes the common issue of rapid aging in carbon membranes. Mechanistic investigations reveal that O_2_ pyrolysis environment selectively removes relatively weakly-bound carbon species, altering pyrolysis intermediates, resulting in a nitrogen-rich framework with disordered nanodomains and heterogeneous ultramicroporosity. This work advances the material chemistry of ultrathin carbon membranes, attractive for ultrafast and high-precision molecular-sieving for molecular separation.

## Abstract (web summary)

Researchers develop 10-nm-thick carbon membranes with oxygen-induced multimodal pore structures that enable rapid, selective and thermally stable hydrogen separation.

## Introduction

Efficient gas separation is a key challenge in a variety of industrial applications, such as hydrogen recovery^1^, natural gas purification^2^, biomass gasification^3^, and ammonia production^4^. Membrane-based separation technologies are particularly attractive due to their energy efficiency and operational simplicity compared to conventional thermally-driven methods like cryogenic distillation or pressure swing adsorption^5^. However, the development of membranes that exhibit both high selectivity and permeance remains a significant challenge in membrane research, due to the inherent permeance-selectivity trade-off^4^.

Carbon-based membranes, especially carbon molecular sieve (CMS) membranes, have shown great promise in overcoming this limitation, owing to their thermal stability, mechanical robustness, and tunable chemical and structural properties. Recent advances have brought CMS membrane performance close to industrial separation applications^6^, combining excellent selectivity^7–13^ with increasing maturity in fabrication^14^. Currently, two primary configurations dominate the CMS landscape: supported thin-film composites on robust porous substrates^15–17^ and asymmetric hollow fibers, which provide high packing density and a self-supporting structure^18–22^. Both benefit from well-established manufacturing processes originally developed for industrial-scale polymeric and ceramic membranes, providing significant scalability advantages. However, regardless of their geometry, most CMS membranes feature a 3–10 μm thick selective layer to prevent defect formation during the pyrolysis process^4^, which inherently limits gas permeance. To address this, significant efforts have been made in substrate engineering^7,16,23–25^ and precursor optimization^10,26^ to push the thickness limit into the sub-micron range for higher permeance. For instance, asymmetric microporous alumina supports were demonstrated to prevent precursor intrusion, yielding 125-nm-thick CMS membranes with high H_2_ permeance (~2000 GPU, 1 GPU = 3.35 × 10^−10^ mol m^−2^ s^−1^ Pa^−1^) and H_2_/N_2_ selectivity (73.5)^16^. A dual-layer hollow fiber was reported with selective layer thickness down to 500 nm, yielding attractive CO_2_ permeance (up to ~2500 GPU) and CO_2_/CH_4_ selectivity (up to 37)^26^. While the potential of reducing selective layer thickness to improve permeance is well recognized, fabricating nanometer-thick CMS membranes without compromising selectivity remains a significant challenge.

Carbon nanomembranes (CNMs) represent another emerging class of carbon-based membranes with thicknesses in the nanometer range. Fabricated via controlled monolayer self-assembly followed by electron-induced cross-linking, these membranes can achieve thicknesses as low as 1–2 nm^27–29^. High gas permeance is obtained owing to their nanometric thickness and minimal transport path length. However, low gas-pair selectivity (for example, H_2_/N_2_ selectivity near 10) limits their practical separation application^30^. This performance gap between ultrathin CNMs and micrometer-thick CMS membranes underscores a critical opportunity: to develop ultrathin, carbon-based membranes that combine the high permeance of CNMs with the molecular selectivity of CMS materials. Despite extensive efforts^16,17,24–26^, however, fabricating ultrathin carbon membranes with both high selectivity and structural stability remains an unresolved challenge, largely due to the difficulty in controlling pore architecture and preventing defect formation at the nanometer scale^7,31^.

Herein, we introduce a class of ultrathin (10-nm-thick) carbon membranes, referred to here as d-UCMs, with nanopore architecture engineered via optimized oxidative pyrolysis. This approach demonstrates carbon membranes at 10-nm-thickness scale exhibiting CMS-level selectivity. Unlike conventional oxidative pyrolysis, which typically aims to functionalize the carbon matrix through oxygen doping or pore wall widening^16,25,32,33^, our approach utilizes oxygen molecules as molecular scissors that selectively cleave weakly bound carbon linkages during pyrolysis and alter the pyrolysis intermediates during carbonization. This mechanism created disordered nanodomains and heterogeneous ultramicroporosity within the ultrathin carbon film, enabling ultrafast and highly selective gas separation.

As a result, d-UCMs exhibit enhanced gas separation performance, with H_2_ permeance exceeding 10,000 GPU and H_2_/N_2_ mixture selectivity surpassing 200, significantly surpassing the permeability–selectivity trade-off of polymeric membranes and even outperforming most CMS membranes. Moreover, the membranes exhibit excellent long-term thermal stability, maintaining performance without aging over 7 days of continuous high-temperature operation. Overall, this work introduces a strategy that combines ultrathin carbon membrane synthesis and precise microstructure sculpting, enabling the scalable platform to fabricate stable membranes with high hydrogen permeance and selectivity for gas separation.

## Results

### Synthesis and structure characterization of the ultrathin carbon membrane

**Fig. 1:**
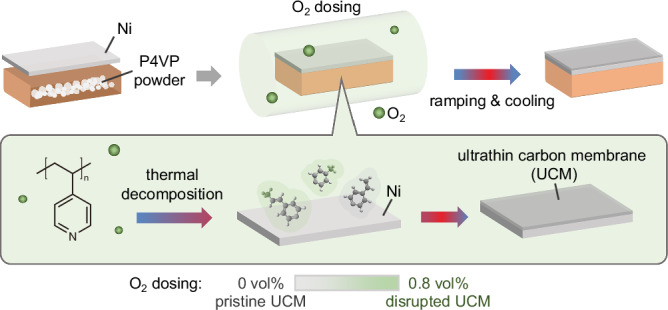
One-step synthesis of pristine and oxygen-disrupted ultrathin carbon membranes (UCMs). Schematic illustration of the one-step synthesis of ultrathin carbon membranes on a nickel substrate, with tunable O_2_ dosing amount from 0 vol% to 0.8 vol%, yielding pristine UCMs (p-UCMs) or disrupted UCMs (d-UCMs).

UCMs were synthesized using a simple, user-friendly, and rapid method (Fig. 1). The pyrolysis environment could be simply regulated by changing the gas environment with a controlled gas dosing system. Poly(4-vinylpyridine) (P4VP) served as the carbon precursor, and nickel served as a substrate for the deposition of an ultrathin carbon film. The synthesis started by simply placing P4VP powder in a boat below a nickel foil. Upon pyrolysis under rapid, atmospheric pressure conditions, volatile decomposition products of P4VP deposited onto a nickel substrate, forming a carbon film.

To investigate the influence of oxidative pyrolysis, a static gas environment with O_2_ dosing (0–0.8 vol%) was created (Supplementary Figs. 1, 2, and Supplementary Note 1). This static gas environment enabled simple control over the composition. A two-stage pyrolysis process was implemented, involving an initial rapid ramp to 400 °C within 25 min, near the decomposition temperature of P4VP, to promote rapid polymer decomposition, followed by a ramp to 500 °C to facilitate homogeneous carbon layer formation. Following this, the reaction was quenched rapidly. The entire process, including cooling to room temperature, was completed in less than 2.5 h.

**Fig. 2:**
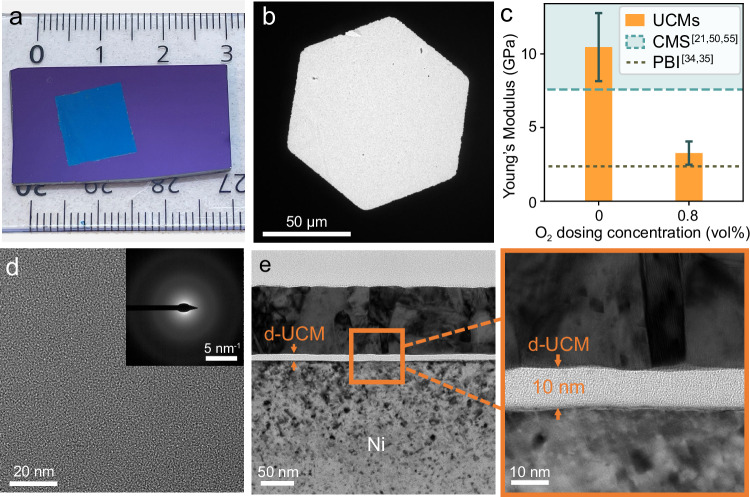
Structural characterization of ultrathin carbon membranes. **a** Optical image of a 1 cm × 1 cm disrupted UCM (d-UCM) film transferred onto a Si wafer. **b** Transmission electron microscopy (TEM) image of a free-standing d-UCM film on a hexagonal copper grid. **c** Elastic modulus of pristine UCM (p-UCM, synthesized with 0 vol% O_2_ dosing) and d-UCM (synthesized with 0.8 vol% O_2_ dosing), compared with hydrogen-selective polymeric films (polybenzimidazole (PBI)) and carbon molecular sieve membranes reported in the literature^21,34,35,50,55^. Error bars for p-UCM and d-UCM represent standard deviation based on three measurements. **d** Top-view high-resolution transmission electron microscopy (HRTEM) image of the free-standing d-UCM and the corresponding diffraction pattern from the same area. **e** Cross-sectional HRTEM image of the d-UCM on a Ni substrate and the corresponding higher-magnification cross-sectional HRTEM image showing the d-UCM thickness of 10 nm and its amorphous carbon structure.

Two distinct types of UCMs were investigated: the pristine UCMs (p-UCMs) prepared under oxygen-free conditions and the disrupted UCMs synthesized in the presence of oxygen. A 1 cm × 1 cm d-UCM film was transferred to a silicon wafer for optical inspection. The representative optical image (Fig. 2a) shows uniform contrast across the entire area, indicating high film uniformity. Further structural analysis was conducted by transferring the film onto a transmission electron microscopy (TEM) grid. A free-standing film suspended over 100-µm-sized holes indicated that the film is mechanically robust (Fig. 2b). Nanoindentation measurements on the film resting on a silicon wafer revealed that both p-UCM and d-UCM films possess high mechanical strength, with elastic moduli of 10.5 ± 1.9 and 3.2 ± 0.6 GPa, respectively (Fig. 2c, Supplementary Fig. 3, and Supplementary Note 2). The lower Young’s modulus of the d-UCM suggests higher porosity of the d-UCM. Nonetheless, the high elastic modulus, comparable to that of polybenzimidazole (PBI, ~2 GPa^34,35^), a state-of-the-art polymeric membrane for H_2_ separation, makes UCMs suitable for integration into a practical membrane.

Selected-area electron diffraction and high-resolution TEM (HRTEM) image (Fig. 2d) revealed that the carbon film is not graphitic but amorphous, with characteristic broad diffuse rings of amorphous carbon. Similar morphology and amorphous structure are also observed in p-UCMs (Supplementary Fig. 4). Cross-sectional analysis of a specimen prepared by focused-ion beam (FIB) using HRTEM revealed the ultrathin nature of the film with a thickness of ~10 nm (Fig. 2e), which was further verified by edge height measurements via atomic force microscopy (AFM) (Supplementary Fig. 5). These ultrathin yet mechanically robust UCMs represent promising candidates for gas separation applications.

### UCMs as highly permeable membranes for gas separation

**Fig. 3:**
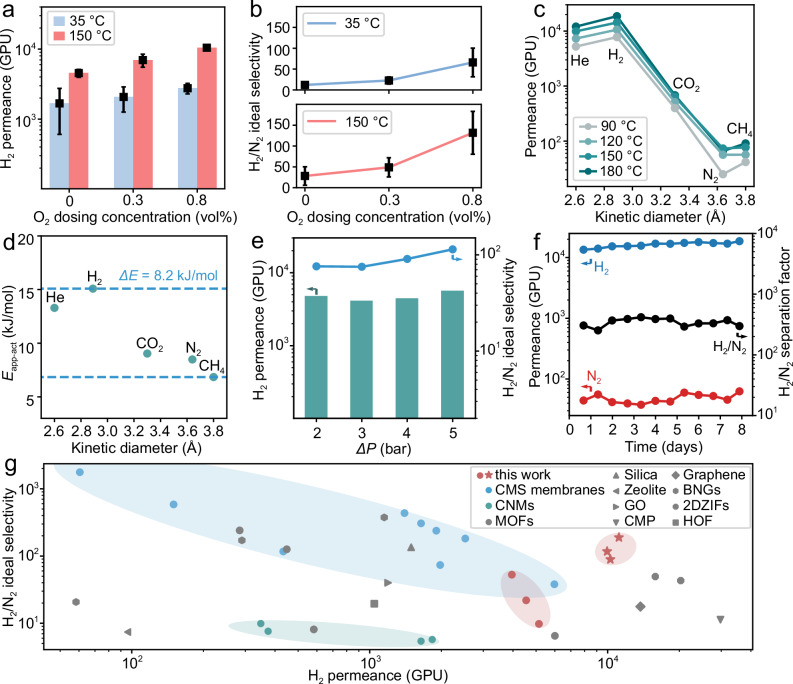
Gas separation performance, transport activation, pressure stability, and benchmarking of ultrathin carbon membranes. Influence of O_2_ concentration during the synthesis on H_2_ permeance in gas permeation units (GPUs) (**a**) and H_2_/N_2_ ideal selectivity (**b**) measured at 35 and 150 °C. Error bars represent the standard deviation from three measurements. **c** Single-gas permeance of He, H_2_, CO_2_, N_2_, and CH_4_ measured at 90, 120, 150, and 180 °C at a feed pressure of 2 bar. **d** Apparent activation energy for gas permeation (${E}_{{{{\rm{app}}}}-{{{\rm{act}}}}}$), plotted as a function of gas kinetic diameter of He, H_2_, CO_2_, N_2_, and CH_4_. Dashed lines highlight the values for H_2_ and CH_4_, and $\Delta E$ denotes the difference between them. **e** H_2_/N_2_ separation performance at 60 °C with a single-gas feed at increasing transmembrane pressure difference, $\Delta P$. **f** Stability test of a d-UCM membrane using a 50/50 H_2_/N_2_ gas mixture over 190 h at 150 °C. **g** Comparison of H_2_/N_2_ ideal selectivity and H_2_ permeance, with reported state-of-the-art membranes, including carbon molecular sieve membranes (CMS)^9,16,56–60^, carbon nanomembranes (CNMs)^30^, metal-organic frameworks (MOFs)^61–64^, silica^65^, zeolite^66^, graphene oxide (GO)^67^, conjugated microporous polymers (CMP)^68^, graphene^69^, boron nitride and graphene nanosheets (BNGs)^70^, 2-dimensional zeolitic imidazolate frameworks (2DZIFs)^71^, hydrogen-bonded organic framework (HOF)^72^, and this work (d-UCM), where circles and stars correspond to data at 35 and 150 °C, respectively.

Crack-free UCM membranes could be successfully prepared by transferring UCM from Ni foil onto a macroporous support using a mechanically-reinforcing film (MRF, Supplementary Fig. 6 and Supplementary Table 1). The MRF was a 250-nm-thick film of poly(1-trimethylsilyl 1-propyne) (PTMSP). The UCM membranes were then sealed in a homemade permeation cell to ensure leak-tight gas transport measurements. Single-gas permeation tests of transport study from p-UCM membranes (0 vol% O_2_) exhibited an average H_2_/N_2_ ideal selectivity of ~12 with an average H_2_ permeance of 1700 GPU at 35 °C (Fig. 3a, b). When the temperature was elevated to 150 °C, the average H_2_/N_2_ ideal selectivity and H_2_ permeance increased to 28 and 4500 GPU, respectively. This is typical when temperature-activated transport dominates from subnanometer-sized pores^36^. The standalone MRF layer has negligible H_2_/N_2_ selectivity (Supplementary Table 2), demonstrating that the observed selectivity originates from the carbon films. Compared to the CNM-based membranes for H_2_/N_2_ separation in literature, where permeance was tested at a low transmembrane pressure difference $\Delta P$ (0.11–0.28 bar)^30^, UCM membranes maintained selectivity under pressurized conditions ($\Delta P$ of 2 bar). This makes UCM highly attractive for membrane-based separation.

Oxidative pyrolysis significantly improved the separation performance. As the O_2_ content increased from 0 to 0.8 vol%, both H_2_ permeance and the H_2_/N_2_ ideal selectivity improved simultaneously (Fig. 3a, b). Specifically, the average H_2_ permeance increased from 1700 to 2800 GPU at 35 °C, and from 4500 to 10,500 GPU at 150 °C. Correspondingly, average H_2_/N_2_ ideal selectivity improved from 12 to 66 at 35 °C and from 28 to 132 at 150 °C. The 0.8 vol% case led to the best performance. For example, a membrane exhibited an ideal selectivity of 189 with a H_2_ permeance of 11,145 GPU at 150 °C. To evaluate the separation performance under industrially relevant conditions, binary mixed-gas tests were conducted with varied feed compositions (Supplementary Fig. 7). The resulting mixed-gas permeance and H_2_/N_2_ selectivity remained comparable to single-gas results, which suggests that competitive transport and sorption effects are negligible in the d-UCM membranes. This is consistent with the gas  adsorption data obtained via a quartz crystal microbalance (QCM) for the d-UCM film (Supplementary Fig. 8). The N_2_ adsorption capacity was ~0.25 mmol/g at 2 bar, while the H_2_ adsorption was below the detection limit. These results suggest the weak binding affinity between the gas molecules and the carbon framework. Notably, all membranes exhibited higher H_2_ permeance and higher H_2_/N_2_ selectivity at elevated temperatures, indicating a temperature-activated H_2_ transport. Since the 0.8 vol% case was the most attractive, the temperature-dependent gas transport was studied between 90 and 180 °C (Fig. 3c). The resulting gas permeance, ${P}_{{{{\rm{g}}}}}$, was fitted with the Arrhenius relationship (Eq. 1) to extract the apparent activation energy (${E}_{{app}-{act}}$) for gas transport:

1 $${P}_{{{{\rm{g}}}}}=A\,{e}^{\left(-\frac{{E}_{{app}-{act}}}{{RT}}\right)}$$

where $A$ is the pre-exponential coefficient, $R$ is the universal gas constant, and $T$ is the temperature. Permeance for all gases increased with temperature, with molecules with smaller kinetic diameter (kd), such as H_2_ (kd of 2.89 Å) and He (kd of 2.60 Å) showing stronger temperature dependence with higher ${E}_{{app}-{act}}$. Specifically, the ${E}_{{app}-{act}}$ for H_2_ was the highest (15 kJ/mol). In comparison, larger molecules like N_2_ (kd of 3.64 Å) and CH_4_ (kd of 3.80 Å) exhibited lower activation energies (8 kJ/mol and 7, respectively). This explains the increase in H_2_/N_2_ permselectivity with temperature (Fig. 3d). The lower apparent activation energies for N_2_ and CH_4_ suggest that transport of these larger molecules is likely through a small population of defects, due to their poor accessibility to the ultramicropores^25^.

Permeation experiments indicate that the d-UCM is highly suitable for H_2_/N_2_ separation, especially at elevated temperatures. Pressure-dependent permeation tests at 60 °C demonstrated the d-UCM with high H_2_/N_2_ selectivity and permeance under transmembrane pressure differences $\Delta P$ up to 5 bar (Fig. 3e). A 190-h (over 7 days) continuous permeation test using H_2_/N_2_ gas mixture (50/50) at 150 °C confirmed membrane stability and resistance to aging, with H_2_ permeance above 10,000 GPU and H_2_/N_2_ mixture selectivity above 200 (Fig. 3f). This combination of thermal stability and high separation efficiency positions UCMs as attractive for hydrogen purification in ammonia cracking and related industrial applications^37,38^. This class of ultrathin carbon membranes with enhanced gas separation performance effectively bridges the performance gap between CMS and CNMs while showing competitiveness against other organic and inorganic membranes (Fig. 3g).

### Oxygen-induced multimodal ultramicroporous structure in ultrathin carbon membranes

Mechanistic studies were conducted to elucidate the structural differences responsible for the enhanced performance of d-UCMs prepared in the presence of O_2_. Two potential roles of O_2_ can be: (1) it acts as a chemical dopant, and incorporates into the carbon membrane structure, potentially shrinking pore sizes or modifying the carbon matrix to enhance selectivity, or (2) it influences the pyrolysis or changes the pyrolysis products^39,40^, leading to structural changes in the pyrolyzed film. To investigate this, we characterized and compared the properties of p-UCM (0 vol% O_2_) and d-UCM (0.8 vol% O_2_).

**Fig. 4:**
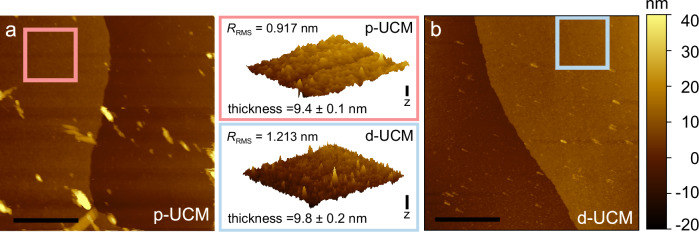
Surface morphology comparison between p-UCM and d-UCM films by atomic force microscopy (AFM). AFM images of the p-UCM film (**a**) and the d-UCM film (**b**) on a Si wafer, together with the corresponding 3D surface profiles of the boxed regions used for root mean square roughness analysis. The pink box corresponds to the selected region in p-UCM, and the blue box corresponds to the selected region in d-UCM. The root mean square roughness values (${R}_{{{{\rm{RMS}}}}}$), film thicknesses, and thickness standard deviations are indicated in the figure. The color gradient represents surface height. The scale bars in the AFM images are 1 μm, while the z-scale bars in the 3D profiles represent 5 nm.

Surface morphology and thickness analyses of the membranes via AFM showed that both p-UCMs and d-UCMs share nearly identical thicknesses (~10 nm) and comparable surface roughness (Fig. 4 and Supplementary Fig. 5). The root mean square roughness measured over a 0.78 μm × 0.78 μm area was similar for p-UCM (0.9 nm) and d-UCM (1.2 nm). These findings highlight that oxidative pyrolysis does not significantly modify the film morphology or thickness, which rules out the possibility that the higher gas permeance observed in d-UCMs is due to a thinner selective layer or a morphology change.

**Fig. 5:**
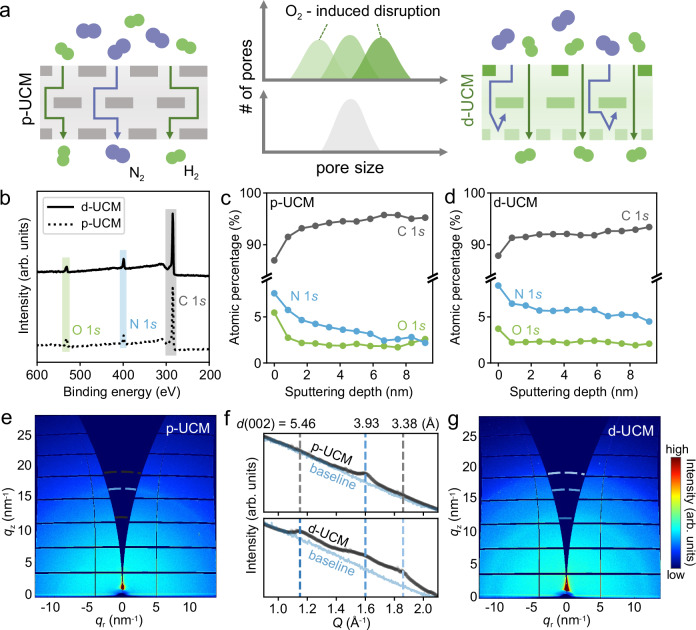
Structural and chemical modifications in UCM films induced by oxidative dosing. **a** Conceptual illustration of the structural evolution in UCM films where oxidative pyrolysis leads to a disrupted and more disordered carbon framework (as indicated by d-spacing shifts) and thus changing the gas permeation behavior. **b** Survey X-ray photoelectron spectroscopy (XPS) on p-UCM and d-UCM. In-depth XPS analysis showing the evolution of C 1*s*, N 1*s*, and O 1*s* concentrations with increasing sputtering depth for **c** p-UCM and **d** d-UCM. Two-dimensional grazing-incidence wide-angle X-ray scattering (2D-GIWAXS) patterns for **e** p-UCM and **g** d-UCM with the corresponding **f** one-dimensional wide-angle X-ray scattering (1D-WAXS) profiles, with dashed vertical lines indicating the characteristic d-spacing positions. The color bar indicates the relative scattering intensity in the GIWAXS patterns.

High-resolution X-ray photoelectron spectroscopy (XPS) was carried out to understand the differences in chemical environment between p-UCM and d-UCM. N 1*s*, and O 1*s* signals were identified, in addition to C 1*s* signal, with sources of N and O being P4VP and O_2_ in the pyrolysis environment, respectively (Fig. 5b). Initial surface scans revealed nearly identical elemental compositions and bonding environments between the p-UCM and d-UCM films (Supplementary Fig. 9), suggesting either a true surface similarity or the masking of underlying differences by ambient contamination. To rule out potential surface contamination effects and further probe the elemental distribution into the material, depth-profiling XPS was performed (Fig. 5c, d). The first point was collected on the as-synthesized surface of p-UCM and d-UCM, where the highest O concentration was observed (5.5 and 3.7% for p-UCM and d-UCM, respectively). Immediately after sputtering, the oxygen concentration decreased to 2% in both films and remained constant throughout the bulk material. Despite the inherent limitations of Ar-ion sputtering, such as potential preferential sputtering or redistribution of light elements, the results provide a reliable qualitative comparison between the two samples. The consistently low and identical oxygen concentrations for p-UCM and d-UCM in their bulk confirm that O_2_ exposure does not result in O incorporation, thereby effectively excluding O_2_ as a dopant.

In contrast, nitrogen and carbon concentrations exhibited distinct differences between the two films. Nitrogen content was consistently higher in d-UCM, both at the surface (7.6 and 8.4% for p-UCM and d-UCM, respectively) and in the bulk (3.5 and 5.6%, respectively). Given the absence of an external nitrogen source during pyrolysis, the increase in nitrogen concentration reflects a relative loss of carbon in the film. This shift in elemental composition suggests that oxygen selectively removes carbon during pyrolysis, enriching the remaining structure in nitrogen. This is further supported by the elevated CO_2_ evolution observed under oxidative pyrolysis, as discussed later in the thermogravimetric analysis coupled with mass spectrometry (TGA-MS) analysis. Building on these results, we propose that oxygen regulates the membrane microstructure during pyrolysis rather than acting as a dopant, prompting a closer investigation of structural features.

Synchrotron-based two-dimensional grazing incidence wide-angle X-ray scattering (2D-GIWAXS) provided further insight into the structural effects of O_2_ modification. Given the ultrathin nature of the membrane, the synchrotron beam is very helpful as it can probe a large area (6–8 mm^2^, Supplementary Fig. 10 and Supplementary Note 3) by using a small grazing angle with the high-intensity synchrotron beam. A single diffuse ring at d(002) = 3.9 Å was observed in p-UCM (Fig. 5e–g), which is significantly larger than that of well-ordered graphite (3.4 Å), reflecting the expanded and disordered nature of carbon domains^41–43^. The diffusivity of the ring suggests the presence of small, randomly oriented carbon domains^44^. In contrast, d-UCM exhibited a more complex structure with a broader diffusive ring with three featuring rings at 3.4, 3.9, and 5.5 Å (Fig. 5g). This broad distribution of interlayer spacings indicates increased structural heterogeneity, which further supports the role of O_2_ in disrupting the carbon network and promoting the formation of a multimodal ultramicroporous structure.

This multimodal ultramicroporous structure of d-UCMs explains the observed high H_2_/N_2_ separation performance. Unlike O-doping strategies typically used for CMS membranes, where pore networks become narrow and where permeance is sacrificed for selectivity, d-UCM synthesis in the oxygen environment leads to a multimodal ultramicroporous structure, improving both permeance and selectivity. Tightly packed carbon regions (d-spacing of 3.38 Å) represent compact structural motifs that correlate with enhanced size-sieving transport, consistent with the preferential permeation of H_2_ (kd of 2.89 Å) over large N_2_ molecules (kd of 3.64 Å). Adjacent broader disordered domains (d-spacing of 5.46 Å) indicate more open structural motifs associated with low-resistance diffusion pathways. This combination of selective sieving and rapid transport allows d-UCMs to achieve significantly enhanced permeance while retaining high selectivity.

**Fig. 6:**
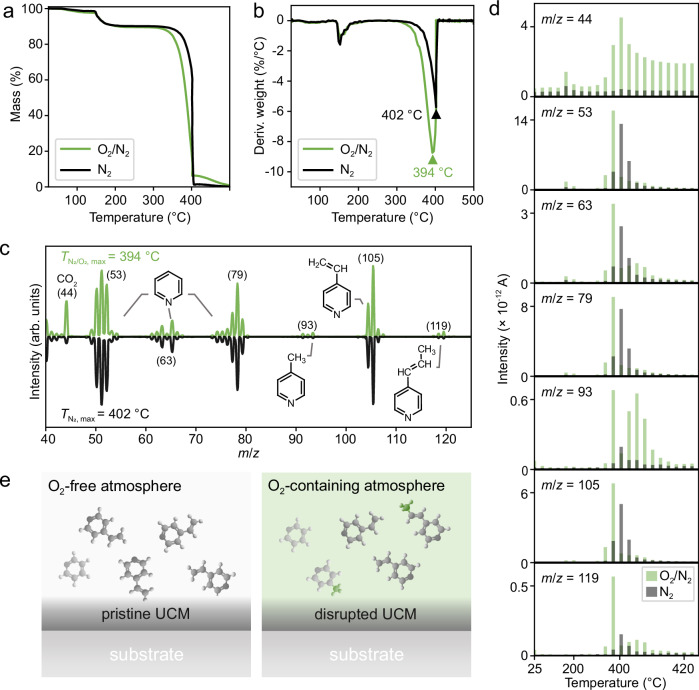
Pyrolysis behavior and decomposition pathways of poly(4-vinylpyridine) (P4VP) in oxidative and oxygen-free atmospheres. **a** Thermogravimetric analysis (TGA) curve of P4VP pyrolyzed in O_2_/N_2_ and N_2_ atmospheres, showing weight loss as a function of temperature. **b** Derivative thermogravimetric (DTG) curves of P4VP highlighting the rate of weight loss and the maximum decomposition temperature (${T}_{\max }$) in different atmospheres. **c** Mass spectra from TGA-MS analysis at ${T}_{\max }$, showing the major pyrolysis products with their corresponding chemical structures and their $m$/$z$ values labeled in brackets. **d** Evolution of MS signal intensity as a function of temperature for major decomposition products, including pyridine ($m$/$z$ = 73–79), methylpyridine ($m$/$z$ = 86–88), vinylpyridine ($m$/$z$ = 104–106), and propenylpyridine ($m$/$z$ = 117–119) under O_2_/N_2_ and N_2_ pyrolysis conditions. The intensity of each species was obtained by integrating the MS signal over the corresponding $m$/$z$ range, providing a quantitative measure of its total release during pyrolysis. **e** Proposed mechanism for disrupted UCM formation through oxygen-mediated vapor-phase structural modifications.

Further insights into the pyrolysis mechanism come from the decomposition behavior of P4VP tracked by TGA-MS. We studied two pyrolysis environments with the oxygen-free environment under nitrogen flow, and the oxygen environment under a flow of nitrogen/oxygen (Fig. 6a, b). In the oxygen-free atmosphere, the maximum decomposition temperature (${T}_{\max }$) was 402 °C, whereas the introduction of oxygen lowered ${T}_{\max }$ to 394 °C. The MS spectrum of deposition products in N_2_ at ${T}_{\max }$ = 402 °C (Fig. 6c) identified vinylpyridine (*m/z* = 105) and pyridine (*m/z* = 79) as the primary products, along with pyridine fragments in MS (*m/z* = 53, 63), methylpyridine (*m/z* = 93), and propenylpyridine (*m/z* = 119) as minor products, consistent with previous studies on P4VP decomposition in inert atmospheres^45,46^. To confirm these assignments and avoid potential interference from ion fragmentation in the MS, the identification of these volatile species was further cross-validated using gas chromatography-mass spectrometry (GC-MS) (Supplementary Fig. 11). Upon introduction of oxygen, while the yields of pyridine and vinylpyridine remained consistent across atmospheres, a notable increase in CO_2_ (*m/z* = 44), methylpyridine, and propenylpyridine was observed. To further investigate how these decomposition products evolved over temperature in different atmospheres, their MS intensities were tracked from 25 to 420 °C (Fig. 6d), covering the temperature range where most decomposition took place. The results revealed that the evolution of pyridine and vinylpyridine aligned closely with the TGA profiles, with an oxygen-induced shift in ${T}_{\max }$ and increased intensity in their evolution curves. Notably, in the oxygen-containing atmosphere, CO_2_ production began as early as 200 °C, while the yield of extra intermediates, such as methylpyridine and propenylpyridine, increased only during major decomposition around 400 °C. The same trend was further supported by GC-MS quantitative analysis of the condensates collected from two pyrolysis atmospheres (Supplementary Fig. 11). These findings indicate that the oxygen environment increases precursor concentration and increases their availability at lower temperatures.

The distinct carbon intermediates observed in the two atmospheres reveal how O_2_ disrupts carbon structure evolution (Fig. 6e). During p-UCMs synthesis, the dominant intermediates are vinylpyridine (*m/z* = 105) and pyridine (*m/z* = 79), accounting for the predominant structural motif (~3.9 Å) observed in p-UCMs, a feature that is also displayed in d-UCMs due to their shared intermediates. This interpretation is further supported by prior studies on π–π stacking distances of pyridine (~3.6 Å) and vinylpyridine (~4.2 Å) dimers^47,48^, suggesting that the 3.9 Å characteristic spacing arises from stacking interactions between these species. In contrast, TGA-MS analysis of d-UCMs exhibited an abrupt CO_2_ release at ~200 °C and an elevated weight loss rate (6 and 9% for p-UCM and d-UCM, respectively, Fig. 6b), consistent with the higher nitrogen content due to carbon loss detected by XPS. These results suggest that oxidative pyrolysis preferentially removes less-stable carbon fragments, promoting denser carbon stacking (3.4 Å), which is close to the interlayer spacing of graphitic carbon. Meanwhile, the accumulation of larger pyrolysis intermediates in d-UCMs, such as propenylpyridine (*m/z* = 119) and methylpyridine (*m/z* = 93) with extended side chains, disrupts π–π stacking during carbonization^49^, leading to the formation of expanded interlayer spacing beyond 3.9 Å in the UCMs.

These insights advance the mechanistic understanding of the role of O_2_ by revealing that it facilitates side-chain scission while leaving the relatively thermally stable pyridine ring largely intact. This leads to a more disordered carbon framework with multimodal ultramicroporous structure, in contrast with the more uniform structure of p-UCMs. Together, these findings present a complete mechanism showing how O_2_ regulates the carbonization behavior and ultimately sculpts the nanostructure and functionality of the resulting UCMs, offering a strategy for nanostructure engineering in carbon-based membranes.

## Discussion

We report a strategy for synthesizing ultrathin carbon membranes with enhanced hydrogen separation performance via oxidative pyrolysis of P4VP on nickel substrates. The synthesis protocol is simple, involving rapid pyrolysis (<2.5 h) under relatively low temperature (500 °C), as compared to CMS membranes, where pyrolysis is often carried out at 600–1000 °C^11,43,50,51^. Controlled oxygen exposure during pyrolysis selectively removes labile carbon bonds while preserving nitrogen-rich domains, resulting in disordered nanostructures with multimodal ultramicroporous networks. Mechanistic studies combining spectroscopy, microscopy, and gas transport analysis confirm that oxygen acts not as a dopant but as a structural modifier, reshaping the carbon framework through oxidative cleavage and side-chain evolution. This work introduces a general and scalable approach to engineer nanostructure in ultrathin carbon films through atmosphere control, bridging the performance gap between CMS membranes and CNMs. The findings have broad implications for advancing membrane-based separations in hydrogen production, carbon capture, and other gas purification technologies.

## Methods

### Materials

Ni foil (25 μm, 99.9%) was purchased from Goodfellow. P4VP (Mw ~160,000 g/mol), FeCl_3_ (97%), and toluene were purchased from Sigma-Aldrich. PTMSP was purchased from abcr. Hydrogen chloride (32%) and isopropanol (99%) were purchased from Reactolab S.A. Acetone was purchased from Thommen-Furler AG.

### Substrate preparation

The Ni foil substrate was washed and ultrasonicated in acetone and isopropanol for 15 min. After that, the dried Ni foil was annealed in a CO_2_ atmosphere at 1000 °C for 20 min to remove organic contaminations. Then it was annealed at 1100 °C for 2 h in H_2_ atmosphere at 760 Torr. After cooling down, a Cu protection layer was sputtered at the backside of the Ni by sputtering Cu for 800 s, at an average sputtering rate of 12.6 Å/s in Alliance-Concept DP 650, which resulted in a 1 μm-thick protection Cu film.

### Ultrathin carbon membranes synthesis

The ~10 nm selective layer of ultrathin carbon membranes (UCMs) were synthesized using an atmospheric pressure pyrolysis procedure inside a chemical vapor deposition (CVD) furnace. 0.1 g P4VP was placed in a copper boat, and the nickel substrate was placed on top of the copper boat. The assembly was sealed and put inside a quartz CVD tube (inner diameter = 2.1 cm, length = 100 cm), which was then evacuated to remove all gas. To establish the pyrolysis atmosphere, a gas mixture of argon (300 sccm, 5 min, 99% of the atmosphere), hydrogen (7.5 sccm, 1 min, 0.5% of the atmosphere), and varying amounts of oxygen (4 sccm, 1 min, and 1.5 min O_2_ exposure, corresponding to 0.3 vol% and 0.8 vol% of the atmosphere) was introduced into the system to reach atmospheric pressure (Supplementary Figs. 1 and 2). After conditioning the atmosphere, all gas flows were halted, and the sample was heated in two stages: first from 25 to 400 °C at a rate of 15 °C/min over 25 min, followed by a slower ramp from 400 °C to 500 °C at a rate of 1 °C/min over 100 min. The temperature was then held at 500 °C for 10 min before the system was rapidly cooled to 25 °C using a compressed air gun.

The resulting carbon film on Ni was coated with 1.5 wt% PTMSP dissolved in toluene by spin coating. A 250-nm-thick layer was formed and left to dry overnight in a clean hood to prepare for subsequent fabrication steps. The PTMSP-coated carbon membrane was floated in 1 M FeCl_3_ solution for 1 h to completely etch away the Ni substrate, allowing the membrane to float on the FeCl_3_ solution. The membrane was then carefully transferred using a glass slide to a 1 M HCl solution for 1 h, followed by rinsing in H_2_O for another 1 h to thoroughly clean the membrane. Finally, the cleaned membrane was transferred onto a macroporous tungsten (W) substrate hosting 5-μm-sized pore arrays as previously reported^25,52,53^ and left to dry overnight under ambient conditions, in preparation for gas permeation measurements.

### Membrane test

The gas permeation properties of the membranes were evaluated using a custom-built permeation setup following our previously reported design^53^. The membrane, supported on a tungsten, was securely sealed within the VCR membrane module (Swagelok) to ensure leak-tight connections.

For single-gas permeation tests, the flow rate for each pure gas (H_2_, N_2_, CO_2_, CH_4_, and He) was maintained at 30 mL/min, with the feed pressure set at 2 bar. Mixed-gas measurements (only for stability evaluation) employed a 50/50 H_2_/N_2_ mixture with a total flow rate of 60 mL/min, corresponding to 1 bar partial pressure for each component at a total feed pressure of 2 bar. The permeate side was swept with argon at a controlled flow rate (ranging from 15 to 50 mL/min).

During each measurement, the membrane was first activated at 150 °C and then cooled down to 35 °C for testing. For temperature-dependent gas transport studies, the membrane was first heated to 180 °C and then sequentially cooled to 150, 120, and 90 °C. Permeation data were recorded after reaching steady-state conditions. The permeate stream was then analyzed using a Hiden HPR-20 R&D mass spectrometer to determine the permeation rates of different gases through the membrane.

### Characterization

#### Focused ion beam and TEM lamella preparation

A gold (Au) layer was sputtered onto the UCM on nickel, followed by a carbon (C) layer deposited on the Au to protect the UCM layer from the ion beam damage. Carbon deposition involved 40 min of electron beam deposition at 30 kV, followed by ion beam deposition at 30 kV, with 40 pA for the initial 100 nm and 150 pA for the subsequent 900 nm. Coarse milling and thinning were carried out at 30 kV using a 13 nA ion beam for milling, followed by sequential thinning steps with 6.5, 3, 1.5, and 0.7 nA beams. Final polishing was performed at 5 kV with 30/80 pA ion beams for 10–20 s, ensuring the lamella met the requirements for TEM imaging.

#### Transmission electron microscopy

The UCM sample was prepared by etching away the nickel using the same procedure as described in membrane fabrication and transferred to a clean Cu grid (400 lines/inch, hexagonal holes). The high magnification high-resolution TEM image and diffraction pattern were achieved in Talos F200S G2 and Spectra 200 S/TEM transmission electron microscope at an acceleration voltage of 200 kV. The low magnification image was achieved using an FEI Tecnai G2 Spirit transmission electron microscope at an acceleration voltage of 120 kV.

#### X-ray photoelectron spectroscopy and in-depth XPS

X-ray photoelectron spectroscopy was performed using a Kratos Analytical Axis Supra instrument. Samples were electrically grounded to the sample stage inside the ultra-high vacuum (UHV) chamber. Measurements were conducted using the monochromated *Kα* line of an aluminium X-ray source (1486.6 eV) with the analyzer set at a pass energy of 20 eV. High-resolution spectra for N 1*s*, C 1*s*, and O 1*s* were acquired with a pass energy of 80 eV and a step size of 0.2 eV.

In-depth XPS analysis was performed using 2 keV Ar^+^ ion sputtering across a 2.5 × 2.5 mm^2^ analysis area in cycles of 30 s, enabling depth-resolved elemental composition analysis. The sputtering rate was calibrated using a 10-nm UCM reference film deposited on silicon, yielding a calculated sputtering rate of 0.0238 nm/s. (Supplementary Fig. 12 and Supplementary Note 4)

#### Atomic force microscopy

AFM and nanoindentation measurements were recorded on a Bruker MultiMode 8 AFM instrument with a SCANASYST-AIR probe, featuring a tip radius of 2 nm, a frequency of 70 ± 25 kHz, and a spring constant of approximately 0.4 N/m. For nanoindentation measurements, the radius of the tip and spring constant were calibrated to be 9.394 nm and 0.3869 N/m, respectively. For the Hertzian fitting, 0.25 was used as Poisson’s ratio in this study for amorphous carbon materials^54^.

#### Grazing incidence wide-angle X-ray scattering

Grazing incidence wide-angle X-ray scattering measurements were performed at the Swiss–Norwegian Beamlines (SNBL) at the European Synchrotron Radiation Facility (ESRF). The optimal incidence angles for the membranes used were 0.03° for the p-UCM (without O_2_) and 0.05° for the d-UCM. The synchrotron beam wavelength was 1.04459 Å for membrane measurements, while substrate measurements were conducted using a beam wavelength of 0.72538 Å. These parameters were optimized to probe the structural properties of the ultrathin carbon membranes while minimizing substrate interference.

#### Thermogravimetric analysis-mass spectrometry

TGA-MS was performed using a TG 209 F1 Libra coupling to QMS 403 Aëolos Quadro quadrupole mass spectrometer. Approximately 5 mg of P4VP was loaded into a tared ceramic crucible and pyrolyzed under different atmospheres, including O_2_ (20%)/N_2_ mixtures and pure N_2_, with a flow rate of 20 mL/min. The heating program replicated the membrane synthesis conditions: heating from 25 to 400 °C at 15 °C/min, followed by a slower ramp to 500 °C at 1 °C/min, holding at 500 °C for 10 min, and then rapidly cooling to 25 °C. This setup enabled simultaneous monitoring of mass loss and identification of decomposition products, providing insights into the pyrolysis mechanism and the role of oxygen in the process.

#### Gas chromatography-mass spectrometry

The condensates obtained under two different pyrolysis conditions (with and without O_2_ dosing) were collected and analyzed by gas chromatography–mass spectrometry (GC-MS) to determine their chemical composition. Gas chromatographic analyses were performed using a Thermo Scientific TRACE 1300 gas chromatograph coupled to a TSQ 8000 Triple Quadrupole GC-MS/MS system equipped with an electron ionization (EI) source and a TriPlus RSH autosampler. Separation was achieved on a MEGA-5 MS XII capillary column (5% phenyl-methylpolysiloxane, 30 m × 0.25 mm i.d., 0.25 µm film thickness). Helium was used as the carrier gas in constant flow mode at 1.2 mL/min. Samples were injected in splitless mode (inlet temperature 250 °C, splitless time 1.0 min, injection volume 1 µL).

The GC oven temperature program was as follows: initial temperature 35 °C, held for 3 min, ramped at 15 °C/min to 150 °C, then ramped at 15 °C/min to 250 °C with a final hold of 3 min. The maximum oven temperature was set to 350 °C.

The MS transfer line and ion source temperatures were both set to 250 °C. The mass spectrometer was operated in electron ionization (EI) mode, with data acquired in full-scan mode over the *m/z* range 50–375. Data acquisition and instrument control were performed using Xcalibur software, and compound identification was based on mass spectral matching against reference libraries. Samples were diluted ten times in MeOH before injection.

#### Gas adsorption measurements through quartz crystal microbalance

Gas adsorption of the ultrathin d-UCM film was measured using a QCM with Dissipation monitoring (QCM‑D, open QCM, Italy) setup capable of recording multiple overtones simultaneously inside a vacuum chamber (Supplementary Fig. 13). The UCM sample was transferred onto a 5 MHz gold-coated quartz sensor using the same method as used for the membrane preparation. After drying at 100 °C and evacuating the system to achieve a stable baseline, the chamber was gradually pressurized with the target gas (H_2_ or N_2_) up to 2 bar. The mass change (Δ*m*) due to gas adsorption was calculated from the frequency shift (Δ*f*) using the Sauerbrey equation:

$$\triangle m=-\frac{C\triangle f}{n}$$

where *C* is the mass sensitivity constant and *n* is the overtone number. A control experiment with a bare gold sensor showed negligible frequency change, confirming that the observed signals originated from adsorption on the UCM films.

## Supplementary information

Supplementary Information

Transparent Peer Review File

## Source data

Source Data

## Data Availability

The data supporting the findings of this study are included in the paper and its Supplementary Information files. Source data are provided with this paper.
