# Supplementary material for: Oxygen-induced multimodal ultramicroporous structure in 10-nm-thick carbon membranes for enhanced hydrogen separation
Source: Nat Commun. 2026 May 22;17:6755. doi: 10.1038/s41467-026-73556-5 (PMC13385404; doi:10.1038/s41467-026-73556-5)
Supplement: Supplementary file 1 — Supplementary Information [file 41467_2026_73556_MOESM1_ESM.pdf]

Supplementary information for

Oxygen-induced multimodal ultramicroporous structure in 10-nm-thick carbon membranes for enhanced hydrogen separation

Yueqing Shen[1], Cédric Van Goethem[1], Heng-Yu Chi[1], Yinghui Li[2], Linyi Li[3], Nicole Barber[4], Kuang-Jung Hsu[1], Daniel Ortiz Trujillo[5], Natalia Gasilova[5], Adam Squires[4], Shiqi Huang [3]*, Kumar Varoon Agrawal[1]*

Affiliations:

1 Laboratory of Advanced Separations, École Polytechnique Fédérale de Lausanne (EPFL); Sion, 1950, Switzerland

2 Laboratory of Materials for Renewable Energy, École Polytechnique Fédérale de Lausanne (EPFL); Sion, 1950, Switzerland

3 Department of Chemical Engineering, Centre for Integrated Materials, Processes & Structures (IMPS), University of Bath; Bath, BA2 7AY, United Kingdom

4 Department of Chemistry, University of Bath, South Building, Soldier Down Ln, Claverton Down, Bath BA2 7AY, United Kingdom

5 Mass Spectrometry and Elemental Analysis Platform (MSEAP), École Polytechnique Fédérale de Lausanne (EPFL), Lausanne, 1015, Switzerland

*Corresponding author. Email: kumar.agrawal@epfl.ch and sh3292@bath.ac.uk

Supplementary Note 1. Calculation of gas concentrations in static pyrolysis environment

The gas concentrations in the static pyrolysis environment were calculated based on the system geometry and gas mixing ratios. The quartz reaction tube (inner diameter = 2.1 cm, length = 100 cm) provided a total volume of 346 cm^3 ($V = \pi r^2 L = \pi(1.05\ cm)^2 \times 100\ cm$), with an additional 30 cm^3 dead volume in the gas lines that was accounted for in the flow rate calculations.

For the 0% O_2 case, the gas mixture contained no oxygen, with hydrogen comprising (2.4% × 76 cm^3)/346 cm^3 × 100% = 0.5% of the total volume, and argon making up the remaining 99.5%. In the 0.3% O_2 condition, oxygen was introduced at a concentration of (1.3% × 76 cm^3)/346 cm^3 × 100% = 0.3%, while hydrogen maintained the same 0.5% concentration, resulting in an argon concentration of 99.2%. The 0.8% O_2 case employed a two-stage gas injection protocol, yielding an oxygen concentration of (1.3% × 74 cm^3 + 1.3% × 152 cm^3)/346 cm^3 × 100% = 0.8%, with hydrogen again at 0.5% and argon at 98.7%.

Supplementary Note 2. Young's modulus calculation for UCMs

The Young's modulus was determined using the Nanoscope software (Bruker) by applying the Hertzian fitting method to the force–indentation data. The Hertzian fitting was applied to the retraction segments of the force curves to minimize the influence of adhesion forces. The fitting was based on the following equation:

$$F = \frac{4}{3}\frac{E}{(1-v^2)}\sqrt{R}\,\delta^{\frac{3}{2}}$$

Where F is the applied force, E is the Young's modulus, R is the tip radius, δ is the indentation depth, and v is the Poisson's ratio, where 0.25 is used in this study for amorphous carbon materials (reference 73 in the main text). The AFM tip radius was calibrated using a standard titanium sample and was determined to be 9.394 nm. Three measurement points were collected for both p-UCM and d-UCM samples. The corresponding force–indentation curves and Hertzian fitting results are shown in Supplementary Fig. 3.

Supplementary Note 3. Synchrotron footprint calculation

The synchrotron beam features a rectangle cross-section with dimension $H \times W$ = 37 μm × 124 μm. When incident on the sample at a grazing angle θ, the beam width W remains unchanged, while the projected beam height H increases to $H/\sin(\theta)$ due to the shallow incidence. Given the small values of θ, the resulting footprint of the beam extends significantly to an order of several mm^2.

In this study, incidence angles of 0.03 and 0.05 were used and the corresponding illumination area can be calculated as follows:

For incidence angle θ = 0.03°, the illumination area $A_{\theta=0.03°}$:

$$A_{\theta=0.03°} = \frac{WH}{\sin(\theta)} = \frac{0.124\ \mathrm{mm} * 0.037\ \mathrm{mm}}{\sin(0.03°)} = 8.86\ \mathrm{mm}^2$$

For incidence angle θ = 0.05°, the illumination area $A_{\theta=0.05°}$:

$$A_{\theta=0.05°} = \frac{WH}{\sin(\theta)} = \frac{0.124\ \mathrm{mm} * 0.037\ \mathrm{mm}}{\sin(0.05°)} = 5.32\ \mathrm{mm}^2$$

Supplementary Note 4. Calculation of sputtering rate v in in-depth XPS

A d-UCM reference sample with a thickness of 10 nm (confirmed by AFM) was transferred to a Si wafer and used to estimate the sputtering rate. The same protocol as in the main experiments was applied, using 2 keV Ar^+ ion sputtering with 30 seconds per cycle. The sputtering depth was determined based on the point at which the elemental concentrations (C/Si) reached a 50/50 ratio, corresponding to 14 cycles (420 s total sputtering time). The sputtering rate was then estimated by dividing the film thickness (10 nm) by the sputtering time (420 s), yielding a rate of approximately 0.024 nm/s. This value provides a reliable estimate of the depth achieved under the specified sputtering conditions.

$$v = \frac{\text{UCM thickness}}{\text{sputtering time}} = \frac{10\ nm}{420\ s} = 0.024\ nm/s$$

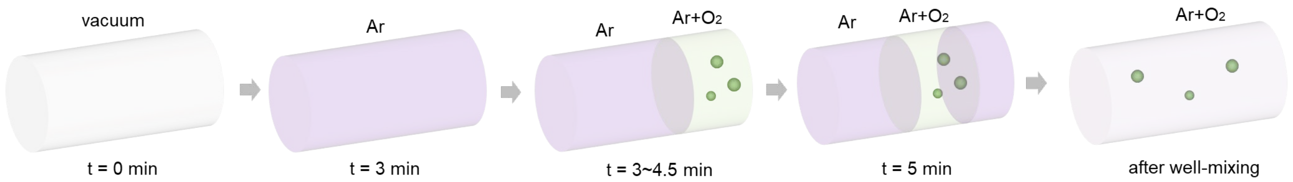

Supplementary Fig. 1. Preparation of the O_2-containing static pyrolysis atmosphere. Illustration of the procedure used to prepare the O_2-containing atmosphere for static pyrolysis.

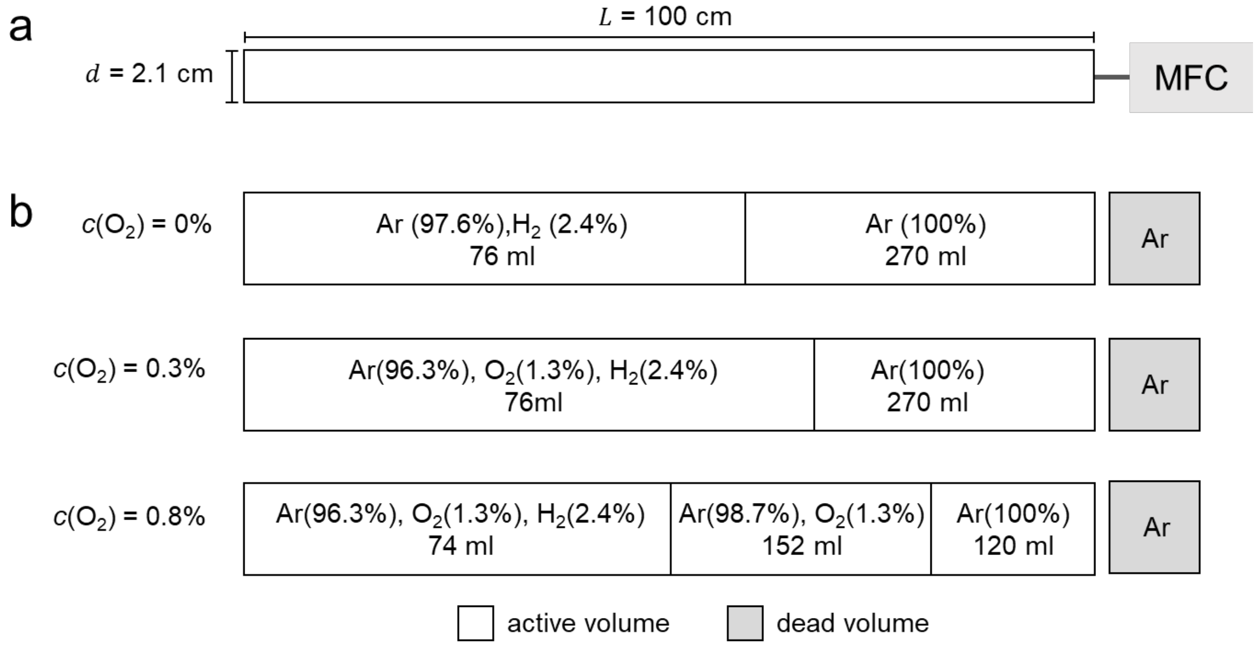

Supplementary Fig. 2. Gas composition in the static pyrolysis atmospheres. (a) Schematic of the experimental setup, including the quartz tube reactor and mass flow controllers (MFCs) used for precise gas mixing. (b) Volumetric distribution of gases used to prepare the three atmospheres with O_2 concentrations of 0%, 0.3%, and 0.8%. The active reaction zone and dead volume within the system are also highlighted.

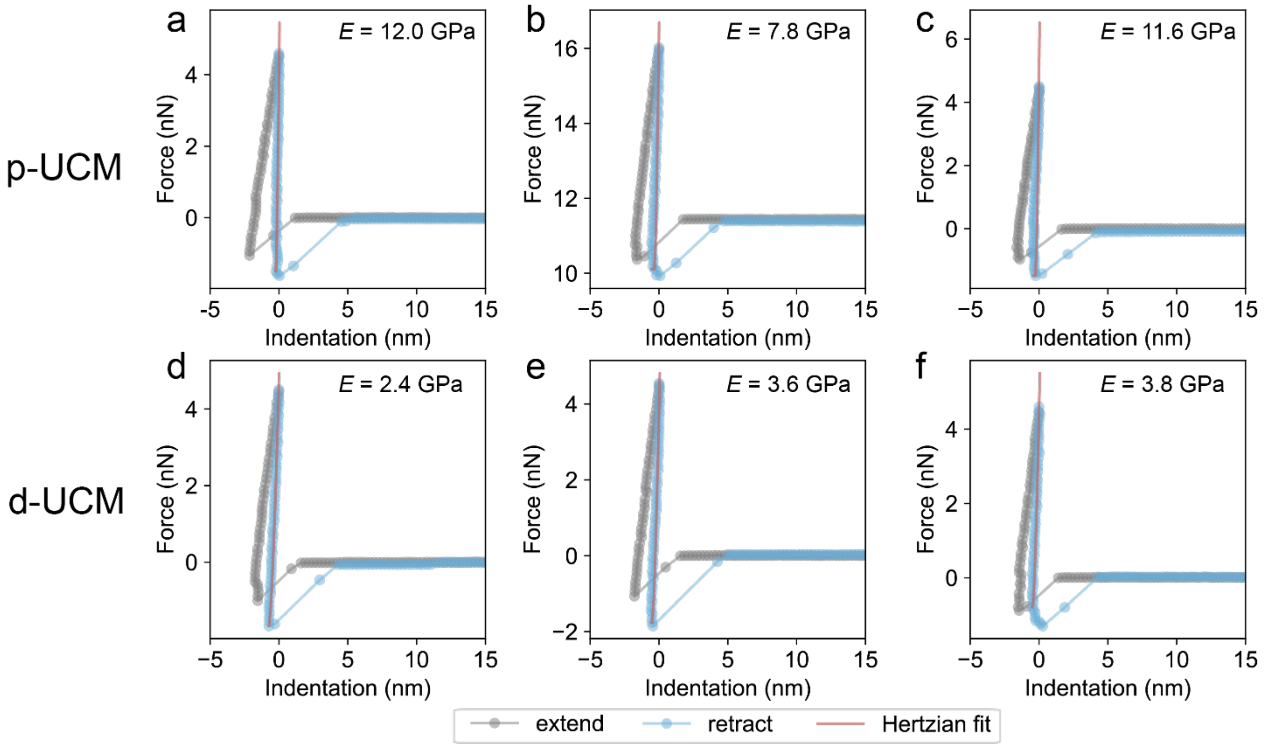

Supplementary Fig. 3. Nanoindentation analysis of p-UCM and d-UCM films. Force-indentation curves and corresponding Hertzian fits for p-UCM (a–c) and d-UCM (d–f), based on the retraction part of the curves. The Young's modulus values were extracted using the Hertz model applied to the retraction data.

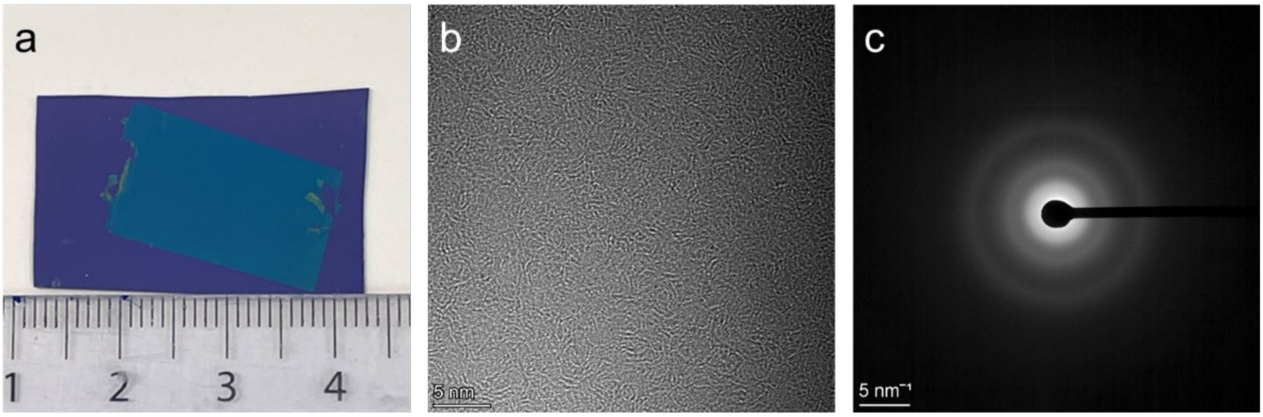

Supplementary Fig. 4. Structural characterization of p-UCM. (a) Optical image of a ~2 cm × 1 cm p-UCM transferred onto a Si wafer. (b) Top-view high-resolution transmission electron microscopy (HRTEM) image of a free-standing p-UCM. (c) Selected-area electron diffraction (SAED) pattern of the free-standing p-UCM.

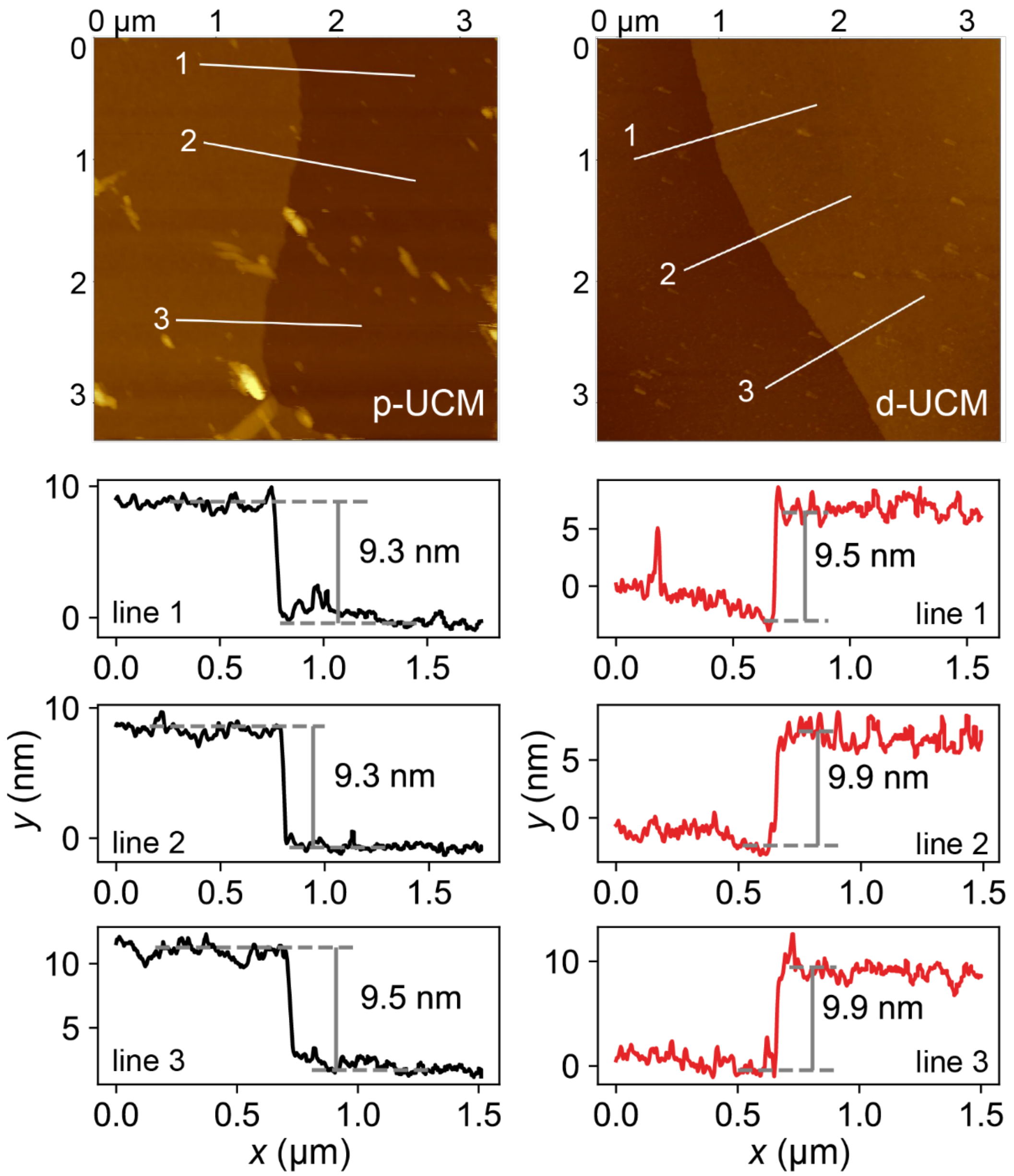

Supplementary Fig. 5. Thickness measurements of p-UCM and d-UCM films by atomic force microscopy (AFM). AFM thickness measurements of p-UCM and d-UCM films, with corresponding line profiles recorded at three different positions for each sample. Black profiles correspond to p-UCM and red profiles correspond to d-UCM.

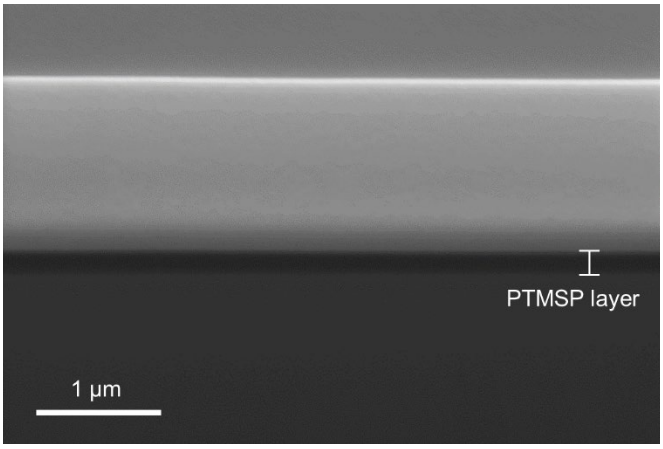

Supplementary Fig. 6. Thickness characterization of the PTMSP mechanically reinforcing film.

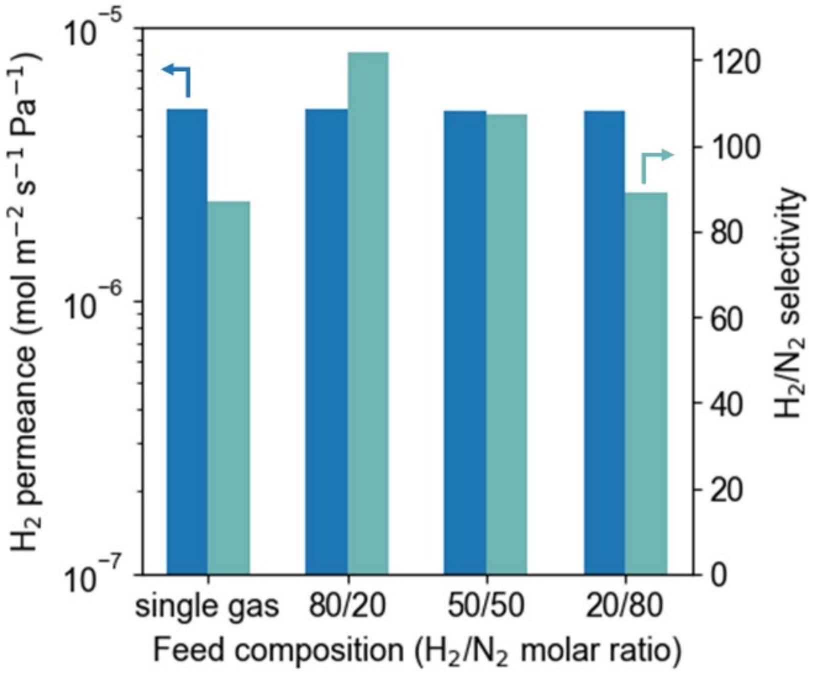

Supplementary Fig. 7. Mixed-gas separation performance of the d-UCM membrane. Gas permeation performance of the d-UCM membrane at 150 °C under single-gas feed and binary-gas feed conditions with H_2/N_2 ratios of 80/20, 50/50, and 20/80.

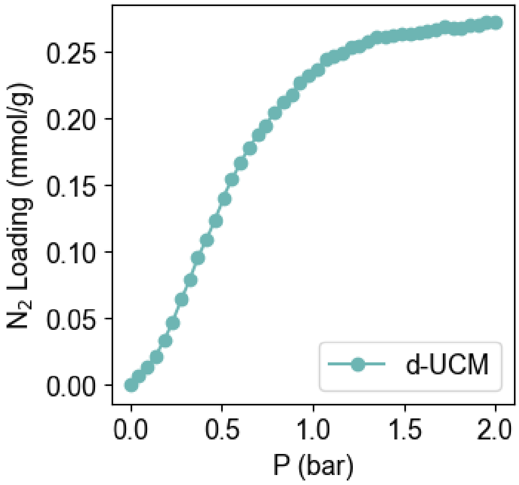

Supplementary Fig. 8. N_2 adsorption at 293 K of the d-UCM film measured by a quartz crystal microbalance setup.

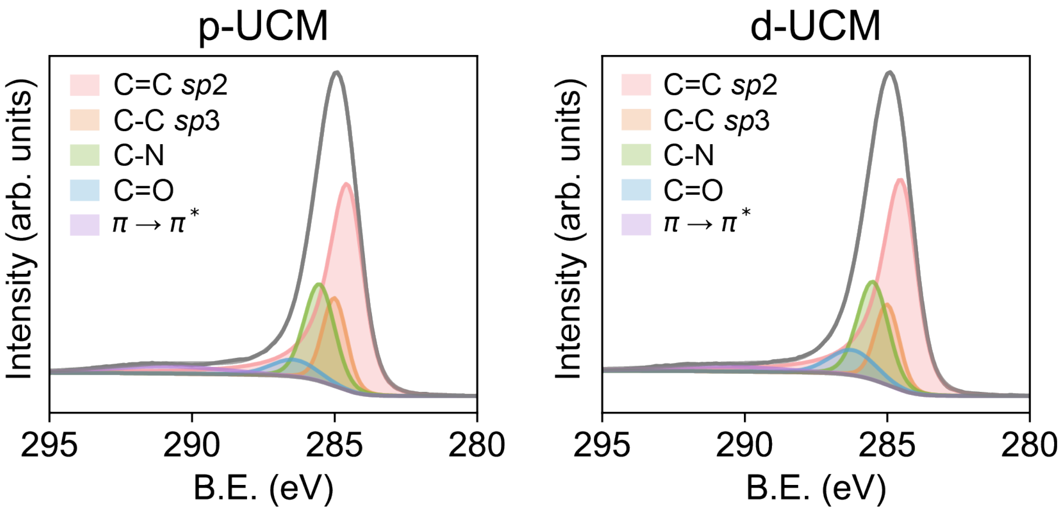

Supplementary Fig. 9. Peak deconvolution of the initial XPS scan showing C 1*s* core level of p-UCM and d-UCM film.

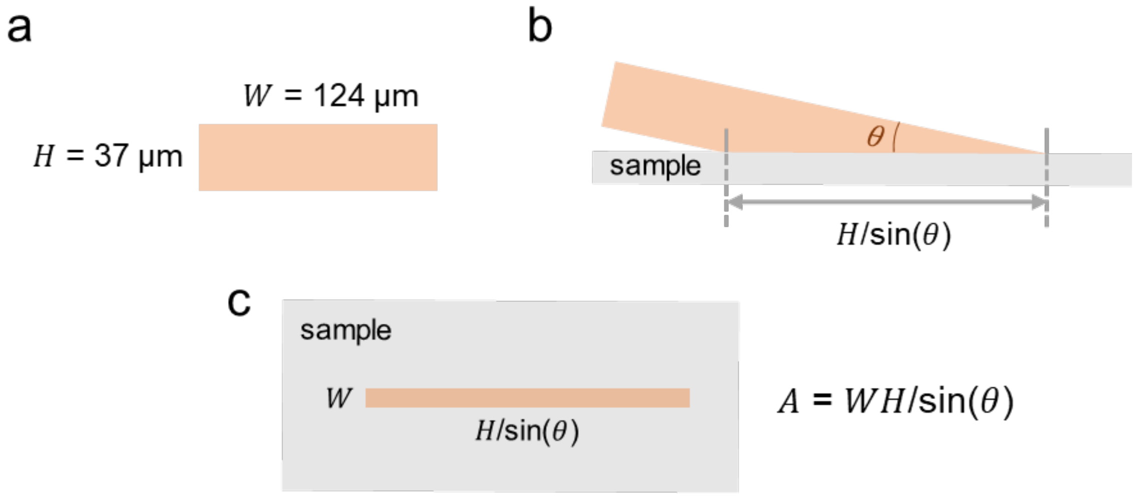

Supplementary Fig. 10. Beam footprint calculation for synchrotron measurements. (a) Size of the synchrotron beam (W = 124 μm, H = 37 μm). (b) Geometric configuration for beam footprint calculation where the incidence angle is θ. (c) Top-view of the beam footprint area (A) on sample surface.

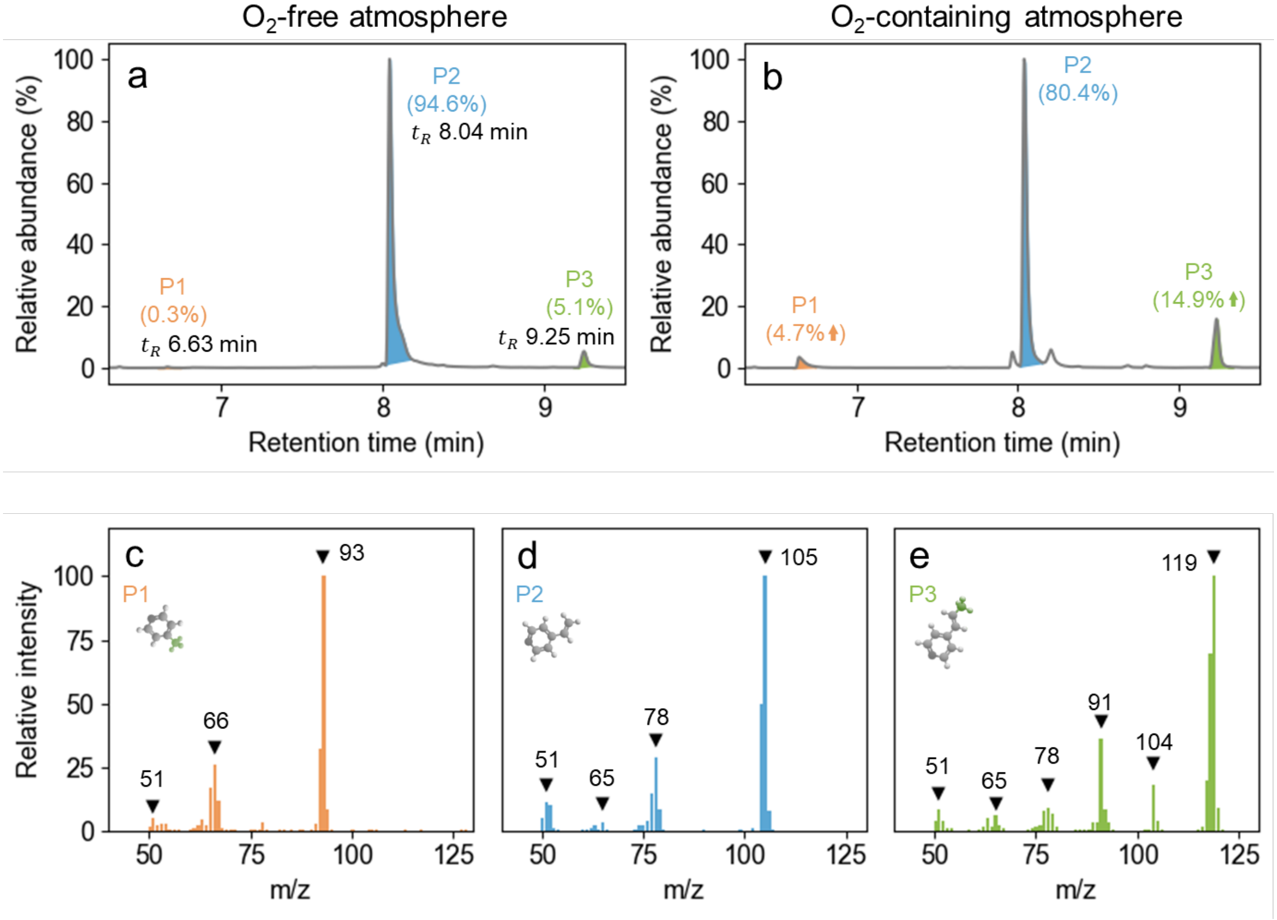

Supplementary Fig. 11. Gas chromatography-mass spectrometry analysis of pyrolysis products. Gas chromatography (GC) traces the pyrolysis products from (a) O_2-free and (b) O_2-containing pyrolysis conditions and the mass spectra of peak at retention time (t_R) 6.63 min, 8.04 min and 9.25 min. With these mass spectra and the NIST database, the molecules are tentatively characterized as methylpyridine at P1, vinylpyridine at P2 and propenylpyridine at P3. GC-MS results quantitatively show that adding O_2 significantly increases the concentration of methylpyridine and propenylpyridine.

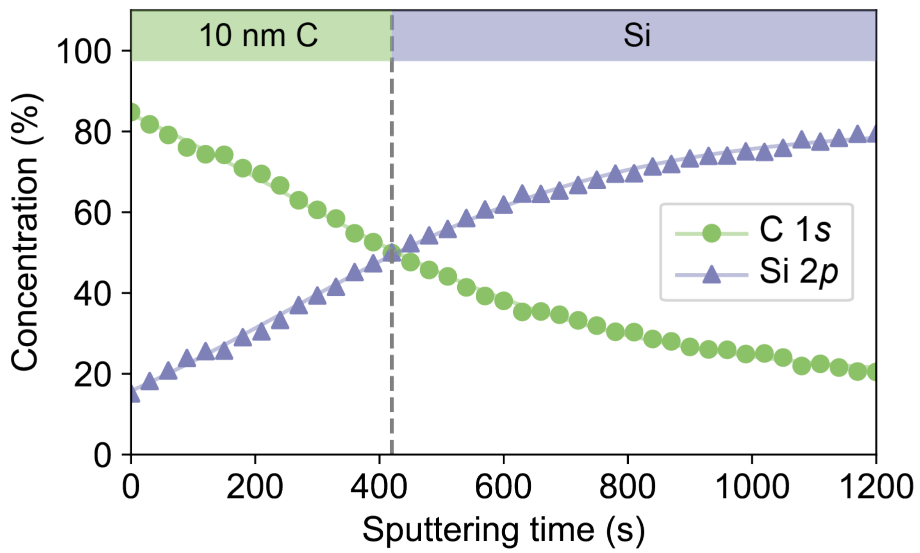

Supplementary Fig. 12. Calibration of the in-depth X-ray photoelectron spectroscopy sputtering rate. In-depth X-ray photoelectron spectroscopy (XPS) calibration test based on a 10-nm-thick UCM transferred onto a Si wafer. The sputtering rate of the UCM was calculated to be 0.0238 nm/s.

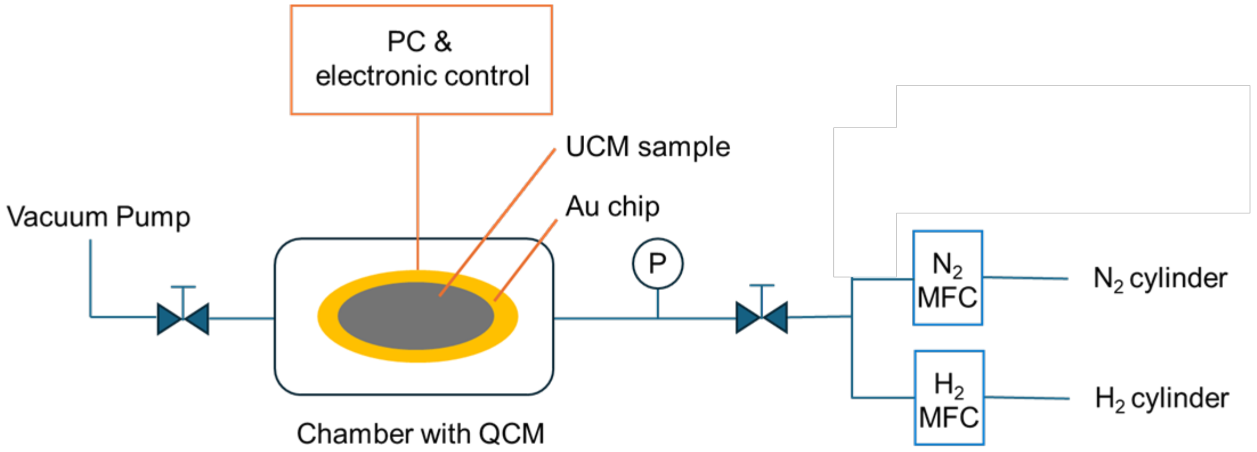

Supplementary Fig. 13. Schematic of the QCM adsorption setup. The ultrathin carbon membrane (UCM) samples are transferred onto a calibrated commercial gold chip. Mass changes during gas adsorption (N_2, and H_2) are monitored by tracking the resonance frequency shifts under controlled vacuum and flow conditions. The detection sensitivity is ~ 0.02 μg.

Supplementary Table 1. Profilometry measurements of the mechanically reinforcing film thickness. Profilometry measurements were performed using a stylus force of 0.1 mg and a stylus tip radius of 2 μm.

Measurement	Thickness (nm)
point 1	235.5843
point 2	228.8524
point 3	268.2167
point 4	240.6715
point 5	286.2711
average	251.9±24.3

Supplementary Table 2. Gas permeance and selectivity of the standalone mechanically reinforcing film. Permeance and selectivity of the mechanically reinforcing film (MRF) at 150 °C and 35 °C, based on three membranes (M1, M2, and M3) measured by single-gas permeation tests. Permeance is reported in gas permeation units (GPU).

	Temp (°C)	He (GPU)	H_2 (GPU)	CH_4 (GPU)	CO_2 (GPU)	N_2 (GPU)	H_2/CH_4	CO_2/N_2	H_2/N_2
M1	150	44246	99432	42697	59750	19470	2.3	3.1	5.1
	35	37466	83044	55798	119068	19342	1.5	6.2	4.3
M2	150	77926	131095	55702	71982	23477	2.4	3.1	5.6
	35	49801	117574	62155	140610	23245	1.9	6.0	5.1
M3	150	75934	134997	56507	76840	23716	2.4	3.2	5.7
	35	47348	109098	61911	148148	22749	1.8	6.5	4.8
