# Supplementary material for: Oxygen-induced multimodal ultramicroporous structure in 10-nm-thick carbon membranes for enhanced hydrogen separation
Source: Nat Commun. 2026 May 22;17:6755. doi: 10.1038/s41467-026-73556-5 (PMC13385404; doi:10.1038/s41467-026-73556-5)
Supplement: Supplementary file 2 — Transparent Peer Review File [file 41467_2026_73556_MOESM2_ESM.pdf]

nature portfolio

Peer Review File

Oxygen-induced multimodal ultramicroporous structure in 10-nm-thick carbon membranes for enhanced hydrogen separation

Corresponding Author: Professor Kumar Varoon Agrawal

This file contains all reviewer reports in order by version, followed by all author rebuttals in order by version.

Version 0:

Reviewer comments:

Reviewer #1

(Remarks to the Author)
The authors fabricated an attractive ultrathin carbon membrane (UCM) with high hydrogen permeance exceeding 10,000 GPU and H_2/N_2 selectivity surpassing 200, maintaining stability for more than 7 days under high-temperature operation. Overall, this manuscript offers valuable insights into O_2-purged pyrolysis, utilizing a relatively simple fabrication method. I recommend it for publication after clarifying and strengthening the following points.
(1) The claim that trace O_2 precisely modulates the carbonization pathway and induces hierarchical ordering is persuasive but remains correlational rather than causal. Also, it is questionable that the O_2 concentration was "precisely regulated" under a static atmosphere. Pyrolysis likely releases volatile organic compounds that alter the internal composition, meaning the actual O_2 partial pressure may differ from the intended value.
(2) The role and justification of the PTMSP reinforcing film require more careful discussion. PTMSP is known to suffer from physical aging and limited mechanical robustness, which can alter its transport behavior. While the authors refer to it as a mechanically reinforcing film (250 nm thick), no mechanical data (e.g., tensile strength, adhesion, crack statistics) are provided. Additionally, its intrinsic N_2 selectivity and thermal aging behavior at 150 °C may influence the overall transport properties. These aspects should be clarified and quantitatively discussed to validate their claimed role in reinforcement.
(3) The authors conclude that O_2 does not act as a dopant based on XPS depth profiling. However, Ar sputtering can cause preferential sputtering and redistribution of light elements, compromising quantitative accuracy. This limitation should be explicitly acknowledged.
(4) The peak assignment of MS may not be unique and could overlap with other fragments. The authors should support these assignments with complementary GC-MS or NMR.
(5) The permeation data appear to be primarily derived from single-gas tests. To ensure practical relevance, mixed-gas permeation tests with varied compositions and pressures are essential, as competitive sorption and diffusion can significantly alter selectivity. The manuscript should clearly separate ideal and mixed-gas results in both terminology and discussion.
(6) In Figure 3b, the y-axis should be labeled "selectivity" or "ideal selectivity" instead of "separation factor," as these are not equivalent terms.
(7) The apparent activation energy symbol is inconsistent (Eapp–act vs. Eact–app). The legend colors are mismatched in Figure 3 – "blue" should replace "grey" for CMS membranes, and "green" should replace "blue" for CNMs. Also, the color "red" should be corrected to "green" in Figure 6. In supplementary information, Figure S7 contains identical labels C–C sp3 for both orange and green colors. The authors should thoroughly review the figure captions.
(8) RRMS values of 0.9 and 1.2 are not identical, though they are close. The use of the term "identical" is overly definitive and should be replaced with a more measured description, such as "similar" or "comparable."
(9) The interpretation of the GIWAXS results appears somewhat overstated. The observed reflections at 3.4, 3.9, and 5.5 Å are described as evidence of a "hierarchical pore structure" responsible for selective H2 transport. However, GIWAXS primarily captures average interlayer or inter-domain spacings and does not directly resolve the continuous transport pathways or effective pore sizes through which gas molecules diffuse. Consequently, these diffraction features should be discussed as structural motifs correlated with, rather than directly representing, the transport-active free volume.

Reviewer #2

(Remarks to the Author)
This work reported a so-called ultrathin carbon membrane for H2/N2 separation. the topic is interesting, with a very high H2 permeance was reported. the CVD method was introduced to deposite a unltrathin carbon films for testing. the membrane preparation process is different from the literature reported method for the fabrication of carbon membranes. however, it is different to say this can be a real carbon membrane. and the films cannot be used standalone, and very low potential for real applications. the following issues should be addressed to approve the advances of this work.
1. Hierarchical porosity usually refers to a material with multimodal pore PSD (ultramicropore, micropores), however, all the pores reported in this work ranging 3.38-5.5 Å belong to ultramicropores. It is confusing to call hierarchical porosity. I doubt the pristine carbon membranes have a unique pore size of 3.9Å. based on the relatively low carbonization temperature of 500 °C, it is highly expected that the prepared carbon membranes are amorphous, which means they may have existing micropores. Gas sorption measurement should be included instead of only with WAXS.

2. The membrane preparation process is way too complicated compared to other types of carbon membranes reported in the literature. The high gas permeance is dependent on the ultrathin carbon layer of 10nm. However, this makes the upscaling extremely challenging (how the authors expect to scale up the CVD process). From fig. 2A, the prepared carbon membranes have an area of 2 cm2 (actually, not the whole membrane can be used for testing). The authors need to clarify the actual membrane area for gas permeation testing. One critical issue is, compared to supported carbon membranes and hollow fiber carbon membranes (a thorough literature review should be included). How would this membrane be scaled up for future industrial applications? As the authors mentioned, the potential for ammonia cracking process where the gas stream pressure is typically 20-40bar tubular reactor or 50-100bar catalytic reactor. How about the membrane stability at higher pressures (more mixed gas permeation testing should be conducted). the stable performance of these physically stable carbon films (on top of the W support) should be measured. This is a critical issue and should be addressed to document the advances of the developed carbon membranes.
3. The mixed gas permeation testing shows higher H2/N2 selectivity compared to that of single gas permeation testing results. The authors need to elaborate more. Both H2 and N2 may have relatively low adsorption capacity (gas sorption experiment should be included).
4. Looking into the hydrogen economy, H2 production from SMR and biohydrogen, as well as H2 recovery from industrial waste gas streams is of great interest. Why the authors believe H2/N2 separation is an urgent issue, which is less challenging compared to H2/CO2 separation. How about the competitiveness of H2/CO2 separation for the developed carbon membranes.
5. For mechanical strength, the authors stated that “The lower Young’s modulus of the d-UCM is comparable to that of polybenzimidazole (PBI), making UCMs suitable for integration into a practical membrane”. A comparison with the literature reported carbon membranes (e.g., PI or cellulose derived (>7.5GPa)) should be included. Instead of comparing with a polymeric membrane.
6. The d-spacing (d(002) detected by WAXS) reflects the distance between carbon planes, not exactly the pores as illustrated in Fig. 5A. The larger d-spacing (compared to Graphite) indicates the amorphous carbon structure. The authors use d-spacing to illustrate that the pore channel of the developed carbon membranes may lead to a misunderstanding for the readers; this must be clarified. Gas sorption measurements using H2 or CO2 should be conducted to characterize the PSD (instead of giving an illustration in Fig. 5a).
7. The prepared film is formed by the deposition of the released small molecules such as propenylpyridine (m/z =119), methylpyridine (m/z = 93), and pyridine and vinylpyridine based on CVD. It’s totally different from the traditional carbon membranes based on the formed carbon plates (mechanically stronger), which means the form films cannot be used standalone, and must be aligned to a support. it might be only for research purposes.

Version 1:

Reviewer comments:

Reviewer #1

(Remarks to the Author)
My comments are well-received, and I think the manuscript can be accepted as it is.

Reviewer #2

(Remarks to the Author)
after carefully reading the revised version, I found that the authors have sufficiently addressed my comments, even though the gas sorption experiment cannot be conducted, the provided alternative way is acceptable. thus, I recommend to publish this work

Open Access This Peer Review File is licensed under a Creative Commons Attribution 4.0 International License, which permits use, sharing, adaptation, distribution and reproduction in any medium or format, as long as you give appropriate credit to the original author(s) and the source, provide a link to the Creative Commons license, and indicate if changes were made.
In cases where reviewers are anonymous, credit should be given to 'Anonymous Referee' and the source.
The images or other third party material in this Peer Review File are included in the article's Creative Commons license, unless indicated otherwise in a credit line to the material. If material is not included in the article's Creative Commons license and your intended use is not permitted by statutory regulation or exceeds the permitted use, you will need to obtain permission directly from the copyright holder.
To view a copy of this license, visit https://creativecommons.org/licenses/by/4.0/

Response to the Reviewer's comments

Reviewer 1

Q 1-1. The claim that trace O2 precisely modulates the carbonization pathway and induces hierarchical ordering is persuasive but remains correlational rather than causal. Also, it is questionable that the O2 concentration was “precisely regulated” under a static atmosphere. Pyrolysis likely releases volatile organic compounds that alter the internal composition, meaning the actual O2 partial pressure may differ from the intended value.

Authors' response: We thank the reviewer for helpful review comments which allowed us to improve the manuscript.

To address the concern that pyrolysis volatiles may alter the internal atmosphere, we estimated our experimental parameters. The sealed pyrolysis tube has a volume of ~0.4 L (containing ~0.016 mol of total gas), whereas the precursor loading is 0.1 g of P4VP. In the extreme scenario where the precursor completely decomposes into vinylpyridine instantaneously (~9.5×10^{-4} mol), this dilution effect will only cause a minor change in the O_2 composition, turning the initial 0.30 vol% O_2 to 0.28 vol% and 0.8 vol% to 0.75 vol%. Therefore, the O_2 concentration does not change significantly.

However, we agree that the term “precisely regulated” may be overly definitive for a static atmosphere. In the revised manuscript, we have replaced “precisely regulated” with “simply” regulated to more accurately describe our experimental approach.

Revised text (Discussion, Section 1, Paragraph 1):
UCMs were synthesized using a simple, user-friendly, and rapid method (**Fig. 1**). The pyrolysis environment could be simply regulated by changing the gas environment with a controlled gas dosing system.

We acknowledge that establishing causality in complex carbonization chemistry is challenging. However, we believe our multi-dimensional evidence helps us to move from correlational observation to mechanistic investigation.

(1) The depth-profiling XPS confirms that oxygen is not incorporated into the bulk carbon lattice, which proves that O_2 acts as a chemical regulator of the carbonization process rather than a reactant or dopant.
(2) The TGA-MS results clearly show that the presence of O_2 directly triggers the early evolution of CO_2 and more importantly enhances the formation of specific side-chain substituted vinylpyridine intermediates.
(3) The GIWAXS data also clearly shows the O_2 induced intermediates disrupt the regular π–π stacking, leading to hierarchical pore structure.

By tracking the process from atmospheric dosing to intermediate gas-phase species, and finally to the resulting lattice spacings, we have established a robust causal framework for oxygen-mediated structural engineering.

Q 1-2. The role and justification of the PTMSP reinforcing film require more careful discussion. PTMSP is known to suffer from physical aging and limited mechanical robustness, which can alter its transport behavior. While the authors refer to it as a

mechanically reinforcing film (250 nm thick), no mechanical data (e.g., tensile strength, adhesion, crack statistics) are provided. Additionally, its intrinsic N2 selectivity and thermal aging behavior at 150 °C may influence the overall transport properties. These aspects should be clarified and quantitatively discussed to validate their claimed role in reinforcement.

Authors' response: We appreciate the reviewer's emphasis on the role and stability of the PTMSP layer. We agree that in composite membranes, the porous support can influence overall transport, particularly if the support undergoes densification or aging that increases resistance.

First, regarding the mechanical role, we want to clarify that PTMSP is not used to improve the tensile strength or stiffness of the membranes. Instead, its primary function is to mitigate capillary-driven stresses during wet processing steps (substrate etching, transfer, and drying)[1,2] to prevent crack-formation in the carbon film during its transfer from the substrate to the porous support.

To quantitatively characterize the mechanical nature of the polymer support, AFM-QNM force-indentation measurements were performed, and Young's moduli were extracted from Johnson-Kendall-Roberts (JKR) fitting (**Figure R1**). The PTMSP layer shows a modulus of ~700 MPa (688±65 MPa). Despite its lower modulus (~700 MPa), the PTMSP layer is ~250 nm thick, an order of magnitude thicker than the carbon film, which then provides a markedly higher fracture energy and strain accommodation capacity. This allows the polymer layer to dissipate capillary stresses during transfer and drying, effectively buffering stress concentrations and preventing crack formation in the ultrathin carbon layer

Second, To address the concern that PTMSP aging or thermal instability may influence the membrane performance, we fabricated membranes using Teflon AF 2400 as an alternative support. We measured the Teflon Young's modulus of Teflon and the performance of the Teflon-supported membrane. As shown in **Figure R1**, the Young's modulus of Teflon (943 ± 134 MPa) is slightly higher than that of PTMSP. As shown in **Figure R2**, the gas permeance and H_2/N_2 selectivity are comparable between the PTMSP-supported and Teflon-supported membranes. This comparison demonstrates that the transport properties are governed primarily by the selective carbon layer, not the support. It also indicates that alternative non-aging polymers can be employed to eliminate aging-related concerns without compromising transport properties.

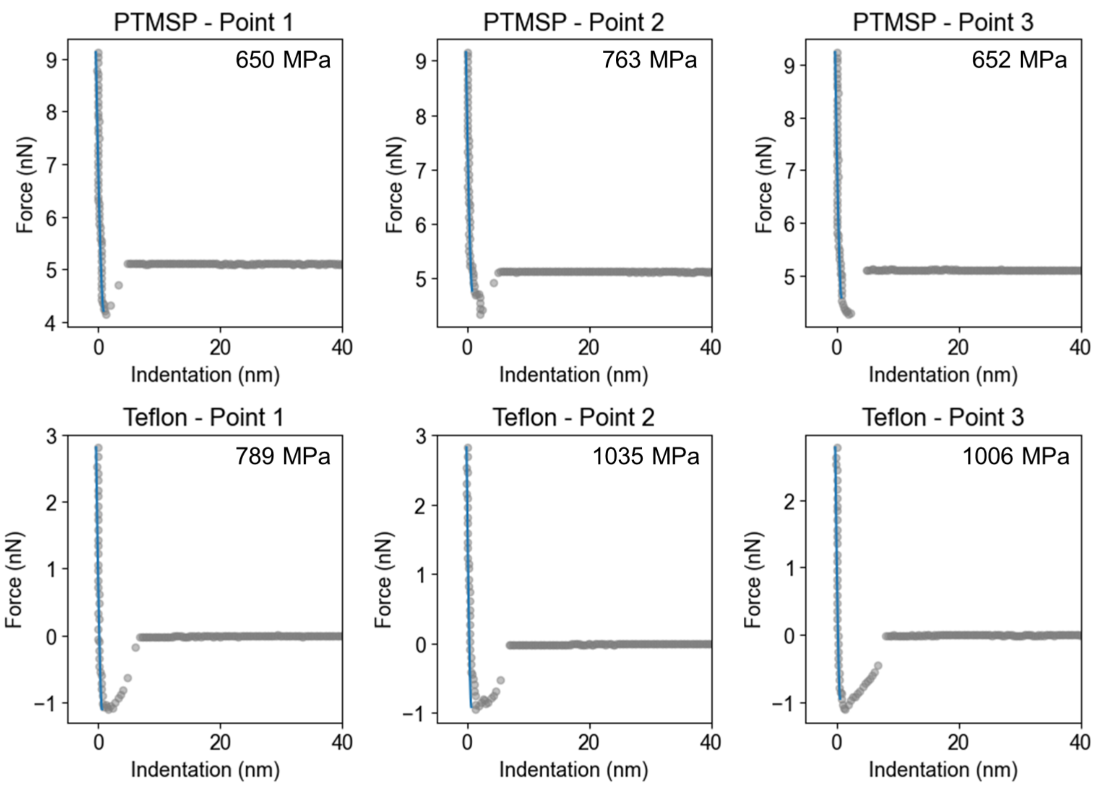

Figure R1. Representative force-indentation curves and corresponding Johnson-Kendall-Roberts (JKR) fits for PTMSP and Teflon support layers based on retraction data. Young's moduli were extracted using the JKR model.

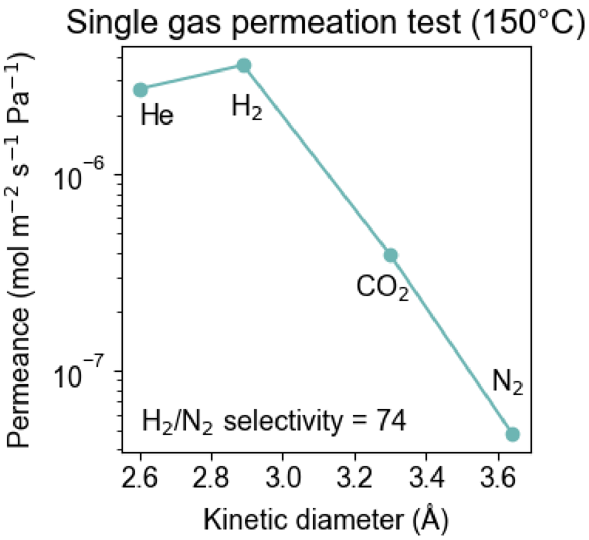

Figure R2. Gas separation performance of the composite membrane fabricated with a Teflon support layer. The permeance and selectivity are from a single gas permeation test at 150 °C.

Q 1-3. The authors conclude that O_2 does not act as a dopant based on XPS depth profiling. However, Ar sputtering can cause preferential sputtering and redistribution of light elements, compromising quantitative accuracy. This limitation should be explicitly acknowledged.

Authors' response: We thank the reviewer for raising this critical point regarding the technical limitations of depth-profiling XPS. We agree that Ar-ion sputtering can indeed

induce preferential sputtering and the redistribution of light elements (such as O), which may compromise the absolute quantitative accuracy of the elemental analysis within the bulk material. We have explicitly acknowledged this limitation in the revised manuscript.

We note that the primary objective of this characterization was to perform a comparative analysis between p-UCM and d-UCM under identical experimental and instrumental conditions.

Revised text

(Discussion, Section 3, Paragraph 3):

Immediately after sputtering, the oxygen concentration decreased to 2% in both films and remained constant throughout the bulk material. Despite the inherent limitations of Ar-ion sputtering such as potential preferential sputtering or redistribution of light elements, the results provide a reliable qualitative comparison between the two samples. The consistently low and identical oxygen concentrations for p-UCM and d-UCM in their bulk, confirm that O_2 exposure does not result in oxygen incorporation, thereby effectively excluding O_2 as a dopant.

Q 1-4. The peak assignment of MS may not be unique and could overlap with other fragments. The authors should support these assignments with complementary GC-MS or NMR.

Authors' response: We thank the reviewer for this critical point. We agree that in TGA-MS, the fragments from pyrolysis and MS ionization can overlap, making it difficult to uniquely identify every species. To address this and confirm our findings, we used complementary NMR, HR-MS, and GC-MS techniques to analyze the pyrolysis condensates.

NMR analysis: We pyrolyzed P4VP under both O-dosed and O-free conditions and collected the volatile condensates for liquid ^{1}H-NMR analysis (**Figure R3** below). The predicted ^{1}H-NMR chemical shifts for main products (methylpyridine, vinylpyridine, and propenylpyridine) are summarized in **Figure R4**, where protons in distinct chemical environments (pyridyl, vinyl, and aliphatic) are highlighted in different colors. By integrating the characteristic regions (**Figure R5**), we observed that the O-dosed sample shows a significantly reduced vinyl-H signal (from 0.34 down to 0.21) and a much higher terminal methyl-H signal (from 0.18 up to 0.37). This clearly indicates that O_2 promotes the conversion of vinyl groups into saturated alkyl-pyridine species (like methyl-pyridine), acting as "molecular scissors" to change the decomposition pathway.

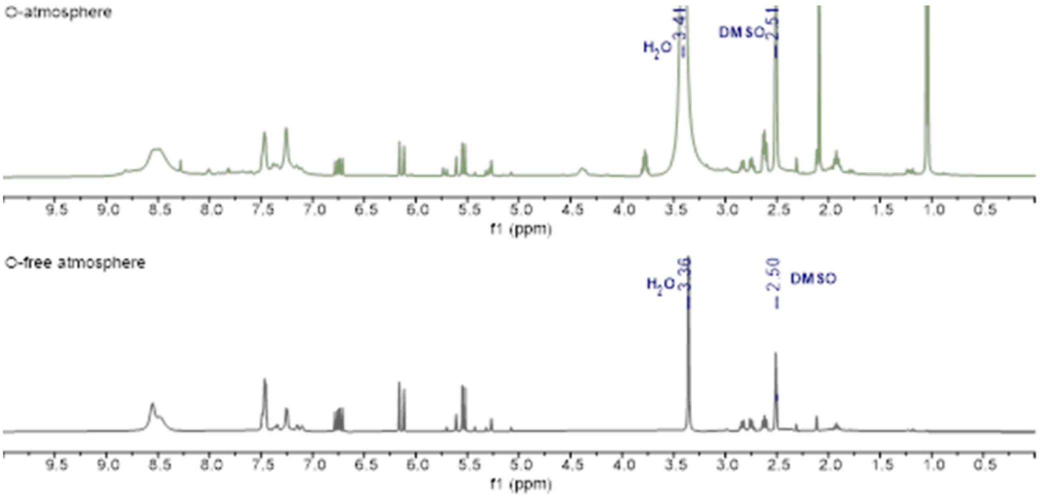

Figure R3. ^{1}H-NMR spectra of condensates collected from P4VP pyrolysis under O_2

dosed condition (O-atmosphere) and O_2 free atmosphere (O-free atmosphere).

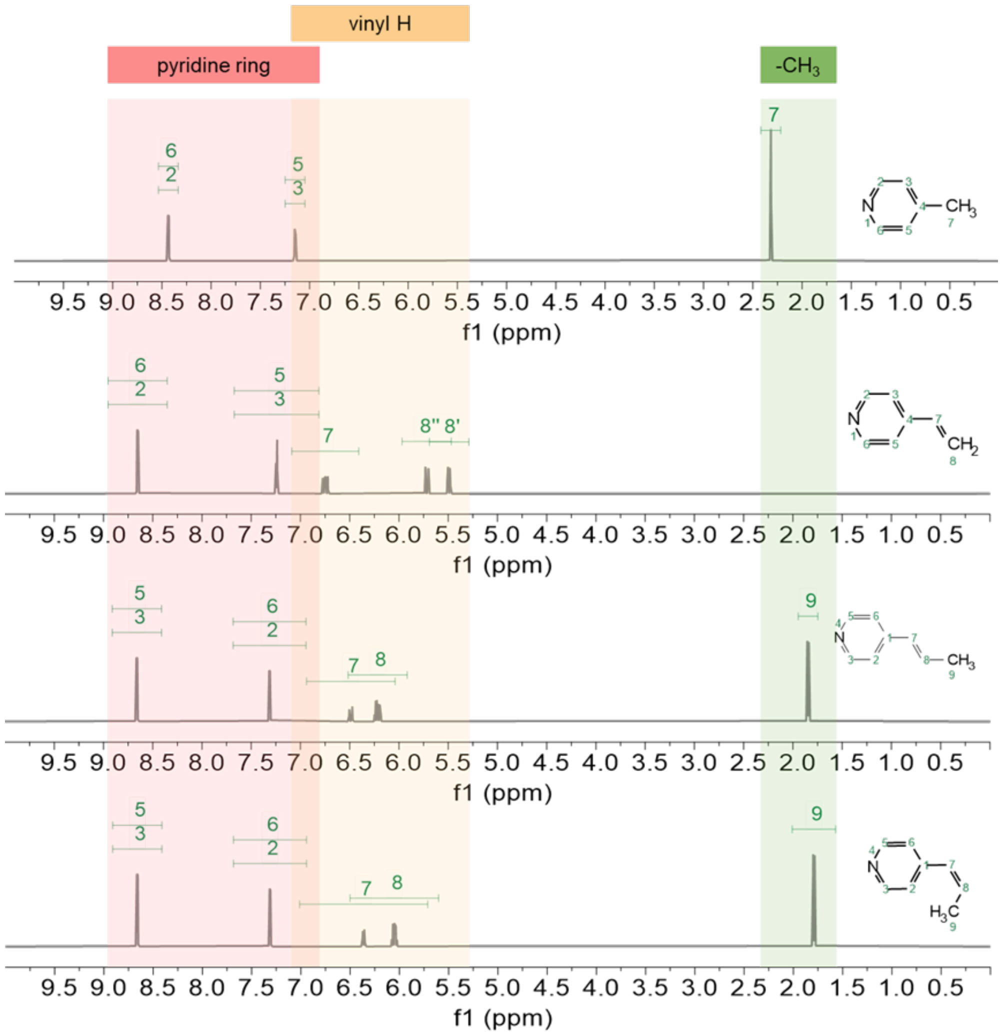

Figure R4. Predicted ^{1}H-NMR chemical-shift ranges of the major pyrolysis products, showing characteristic regions for pyridyl protons, vinyl protons, and terminal methyl protons. Error bars represent the estimated uncertainty in the prediction.

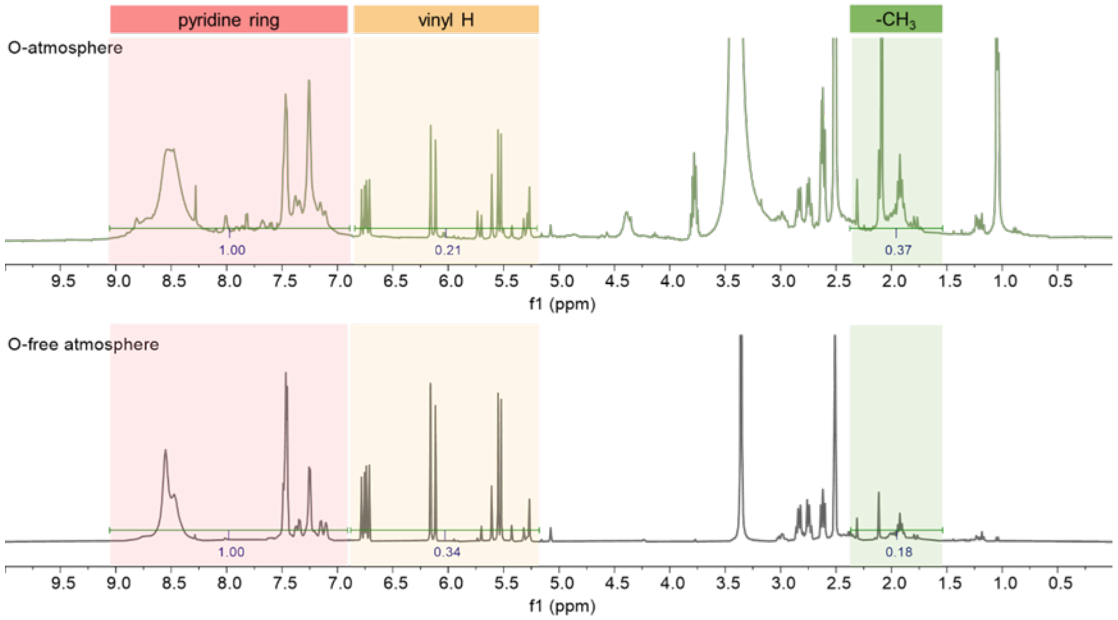

Figure R5. Normalized integrals of the pyridine, vinyl, and methyl proton regions in condensates obtained under O-dosed and O-free pyrolysis conditions.

HR-MS and GC-MS Analysis: To further confirm the specific molecules, we performed HR-MS (**Figure R6**), which uses a soft ionization method to avoid breaking molecules into fragments. This confirmed the presence of methyl-pyridine (m/z = 94), vinylpyridine (m/z = 106), and propenylpyridine (m/z = 120) with one proton protonated $[M+H]^+$.

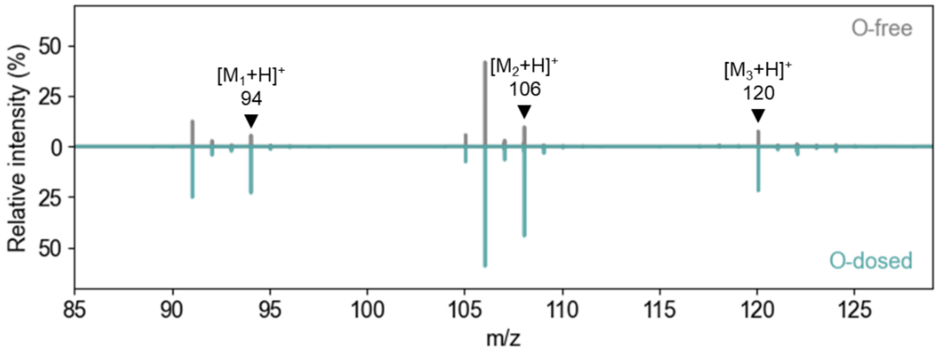

Figure R6. High resolution mass spectra of O-free pyrolysis products (grey curve) and O-dosed pyrolysis products (green curve). The peaks M1, M2, M3 are assigned to protonated [M+H]+ species of methyl-pyridine (m/z=94), vinylpyridine (m/z=106) and propenylpyridine (m/z=120).

Finally, we used GC-MS (**Figure R7**) to separate these components. We clearly identified three distinct peaks at retention time of 6.63, 8.04 and 9.25 min, which correspond to methylpyridine, vinylpyridine, and propenylpyridine according to NIST database. Most importantly, the GC-MS results quantitatively show that adding O_2 significantly increases the concentration of methylpyridine and propenylpyridine.

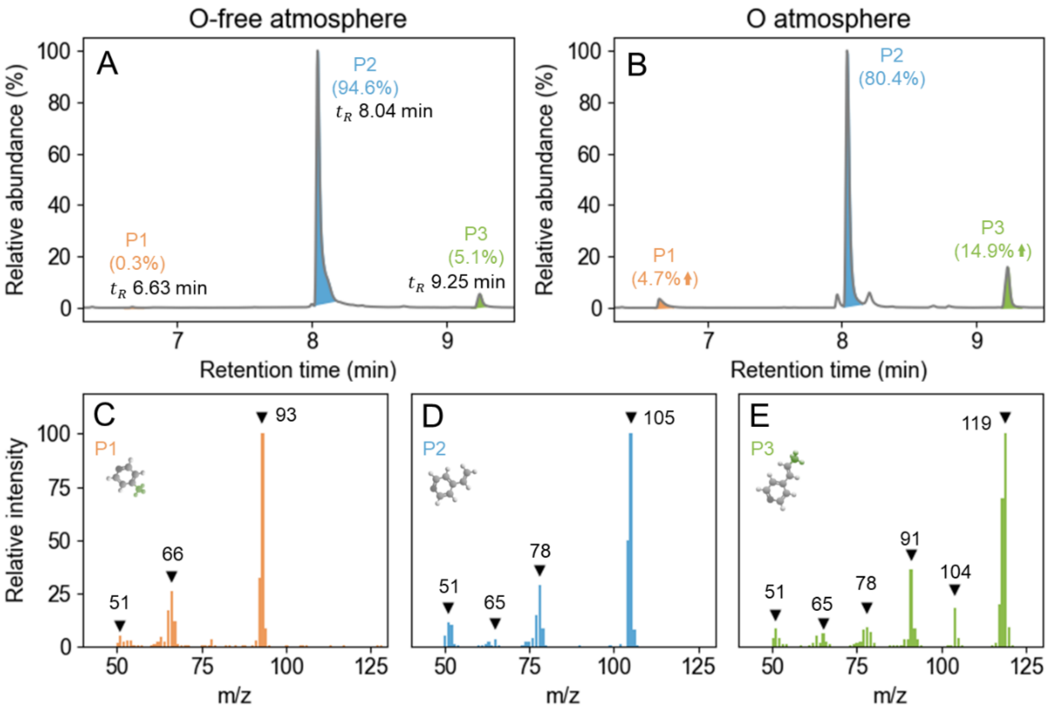

Figure R7. Gas chromatography (GC) of the pyrolysis products from (A) O-dosed and (B) O-free pyrolysis conditions and the mass spectra of peak at retention time (t_R) 6.63 min, 8.04 min and 9.25 min. With these mass spectra and the NIST database, the molecules are tentatively characterized as methylpyridine at P1, vinylpyridine at P2 and propenylpyridine at P3.

These results are consistent with our original TGA-MS observations, confirming that O_2 triggers the formation of these specific substituted pyridine intermediates.

We have added a Figure S11 and revised the main text as follows.

Revised text:
(Discussion, Section 3, Paragraph 7)
The MS spectrum of deposition products in N_2 at T_{max} =402 °C (**Fig. 6c**) identified vinylpyridine (m/z = 105) and pyridine (m/z = 79) as the primary products, along with pyridine fragments in MS (m/z = 53, 63), methylpyridine (m/z = 93) and propenylpyridine (m/z = 119) as minor products, consistent with previous studies on P4VP decomposition in inert atmospheres[65,66]. To confirm these assignments and avoid potential interference from ion fragmentation in the MS, the identification of these volatile species was further cross-validated using gas chromatography-mass spectrometry (GC-MS) (Fig. S11). …The same trend was further supported by GC-MS quantitative analysis of the condensates collected from two pyrolysis atmospheres (Figure S11). These findings indicate that the oxygen environment increases precursor concentration and increases their availability at lower temperatures.

(Methods)
1.5.7. Gas chromatography-mass spectrometry
The condensates obtained under two different pyrolysis conditions (with and without O_2 dosing) were collected and analyzed by gas chromatography–mass spectrometry (GC–MS) to determine their chemical composition. Gas chromatographic analyses were performed using a Thermo Scientific TRACE 1300 gas chromatograph coupled to a TSQ 8000 Triple Quadrupole GC-MS/MS system equipped with an electron ionization (EI) source and a TriPlus RSH autosampler. Separation was achieved on a

MEGA-5 MS XII capillary column (5% phenyl-methylpolysiloxane, 30 m × 0.25 mm i.d., 0.25 µm film thickness). Helium was used as the carrier gas in constant flow mode at 1.2 mL/min. Samples were injected in splitless mode (inlet temperature 250 °C, splitless time 1.0 min, injection volume 1 µL).

The GC oven temperature program was as follows: initial temperature 35 °C, held for 3 min, ramped at 15 °C/min to 150 °C, then ramped at 15 °C/min to 250 °C with a final hold of 3 min. The maximum oven temperature was set to 350 °C.

The MS transfer line and ion source temperatures were both set to 250 °C. The mass spectrometer was operated in electron ionization (EI) mode, with data acquired in full-scan mode over the m/z range 50–375. Data acquisition and instrument control were performed using Xcalibur software, and compound identification was based on mass spectral matching against reference libraries. Samples were diluted ten times in MeOH before injection.

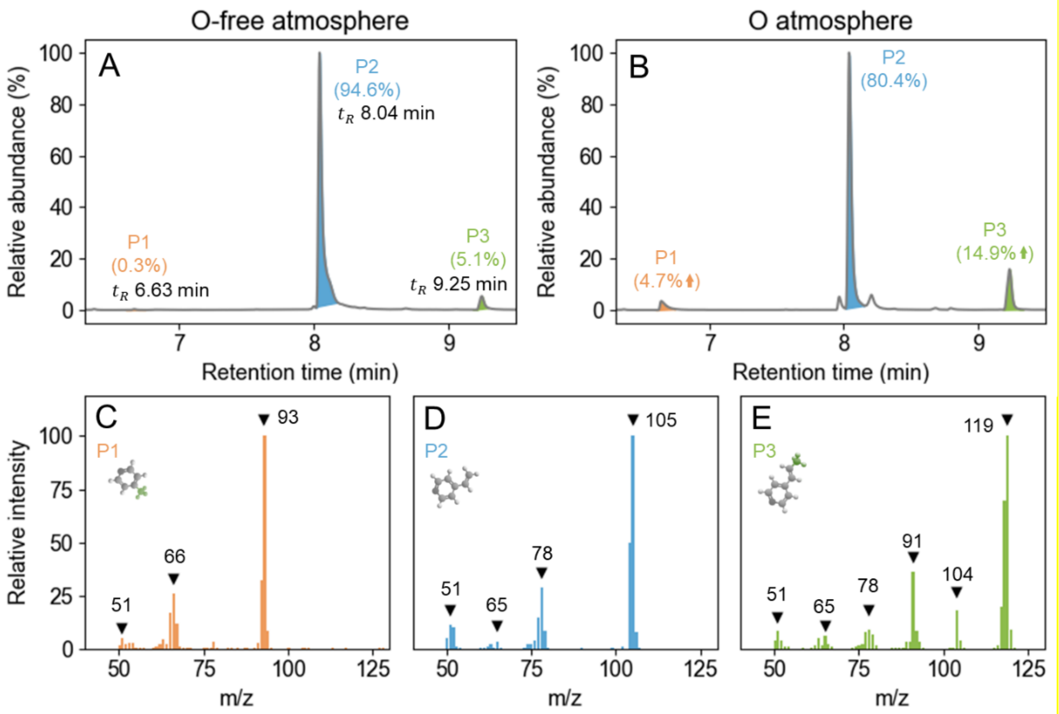

Fig. S11 Gas chromatography (GC) of the pyrolysis products from (A) O-dosed and (B) O-free pyrolysis conditions and the mass spectra of peak at retention time (t_R) 6.63 min, 8.04 min and 9.25 min. With these mass spectra and the NIST database, the molecules are tentatively characterized as methylpyridine at P1, vinylpyridine at P2 and propenylpyridine at P3. GC-MS results quantitatively show that adding O_2 significantly increases the concentration of methylpyridine and propenylpyridine.

Q 1-5. The permeation data appear to be primarily derived from single-gas tests. To ensure practical relevance, mixed-gas permeation tests with varied compositions and pressures are essential, as competitive sorption and diffusion can significantly alter selectivity. The manuscript should clearly separate ideal and mixed-gas results in both terminology and discussion.

Authors' response: We fully agree with the reviewer that mixed-gas permeation tests are essential for evaluating membranes under industrially relevant conditions. As suggested, we have included the binary H_2/N_2 mixed-gas test (**Figure R8**) with varied feed gas composition (H_2/N_2 molar ratios of 80/20, 50/50, and 20/80) at 150 °C, and compare them with the single gas permeation results on the same membrane sample. As shown in the new **Figure R8**, the H_2 permeance in all binary gas tests remains

nearly identical to the single-gas permeance (~4.9×10^{-6} mol m^{-2} s^{-1} Pa^{-1}) while the H_2/N_2 selectivity ranges from 87 to 122. This demonstrates that competitive H_2 transport effects play a minor role in the current work. This is mainly because the transport of hydrogen is in the molecular sieving regime, and hydrogen and nitrogen do not have strong binding affinities in porous carbon. To include this point, we add a brief discussion in the main text and Figure R8 as Fig. S7 in supplementary information.

Revised text:
(Discussion, Section 2, Paragraph 2)
The 0.8 vol% case led to the best performance. For example, a membrane exhibited an ideal selectivity of 189 with a H_2 permeance of 11145 GPU at 150°C. To evaluate the separation performance under industrially relevant conditions, binary mixed-gas tests were conducted with varied feed compositions (**Fig. S7**). The resulting mixed-gas permeance and H_2/N_2 selectivity remained comparable to single-gas results, which suggests that competitive transport and sorption effects are negligible in the d-UCM membranes.

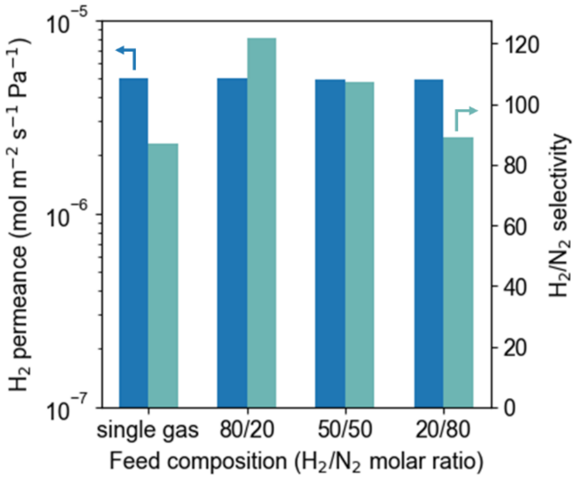

Figure R8 (Fig. S7). Gas permeation test of d-UCM membrane at 150°C with binary feed gas.

Regarding the difference between ideal and mixed-gas separation, we have thoroughly revised the manuscript to ensure that "ideal selectivity" (from single-gas tests) and "mixed-gas selectivity" (from binary tests) are clearly separated in both terminology and discussion. Aside from the long-term stability and mixed-gas composition tests, all other performance data in the test refer to ideal gas permeation.

Revised text:
(Abstract)
This optimized distribution of free volume in a 10-nm thick membrane enables a record combination of H_2 permeance exceeding 10,000 gas permeation units (GPUs) and H_2/N_2 mixture selectivity surpassing 200.

(Discussion, Section 2, Paragraph 1)
Single-gas permeation tests of transport study from p-UCM membranes (0 vol% O_2) exhibited an average H_2/N_2 ideal selectivity of ~12 with an average H_2 permeance of 1700 gas permeation units (GPU) at 35 °C (**Fig. 3a-b**). When the temperature was elevated to 150 °C, the average H_2/N_2 ideal selectivity and H_2 permeance increased to 28 and 4500 GPU, respectively.

(Discussion, Section 2, Figure 3 caption)
Fig 3. Mechanistic evaluation and benchmarking of UCMs based on gas

permeation performance. The influence on the H_2 permeance (**a**) and H_2/N_2 ideal selectivity … (**g**) Comparison of H_2/N_2 ideal selectivity and H_2 permeance (in GPU) among the state-of-the-art CMS membranes for H_2/N_2 separation[9,18,31–35] (blue circles), CNMs[23] (green circles), metal-organic frameworks[36–39] (MOFs, grey circles), silica[40] (grey upward triangle), zeolite[41] (grey leftward triangle), graphene oxide[42] (GO, grey rightward triangle), conjugated microporous polymers[43] (CMP, grey downward triangle), graphene[44] (grey diamond), boron nitride and graphene nanosheets[45] (BNGs, grey hexagons), 2-dimensional zeolitic imidazolate frameworks[46] (2DZIFs, grey octagons), hydrogen-bonded organic framework[47] (HOF, grey square), and this work (d-UCMs, red circles for data at 35°C and red stars, data at 150 °C).

(Discussion, Section 2, Paragraph 2)
Oxidative pyrolysis significantly improved the separation performance. As the O_2 content increased from 0 to 0.8 vol%, both H_2 permeance and the H_2/N_2 ideal selectivity improved simultaneously (**Fig. 3a-b**). Specifically, the average H_2 permeance increased from 1700 to 2800 GPU at 35 °C, and from 4500 to 10500 GPU at 150 °C. Correspondingly, average H_2/N_2 ideal selectivity improved from 12 to 66 at 35 °C and from 28 to 132 at 150 °C. The 0.8 vol% case led to the best performance. For example, a membrane exhibited an ideal selectivity of 189 with a H_2 permeance of 11145 GPU at 150°C.

(Discussion, Section 2, Paragraph 3)
A 190-hour (8-day) continuous permeation test using H_2/N_2 gas mixture (50/50) at 150 °C confirmed membrane stability and resistance to aging, with H_2 permeance above 10,000 GPU and H_2/N_2 mixture selectivity above 200 (**Fig. 3f**).

Q 1-6. In Figure 3b, the y-axis should be labeled “selectivity” or “ideal selectivity” instead of “separation factor,” as these are not equivalent terms.

Authors' response: We thank the reviewer for this correction. We have updated the y-axis label in **Figure 3b** from "separation factor" to "ideal selectivity" to more accurately reflect the single-gas permeation measurements used.

Revised text and figures:

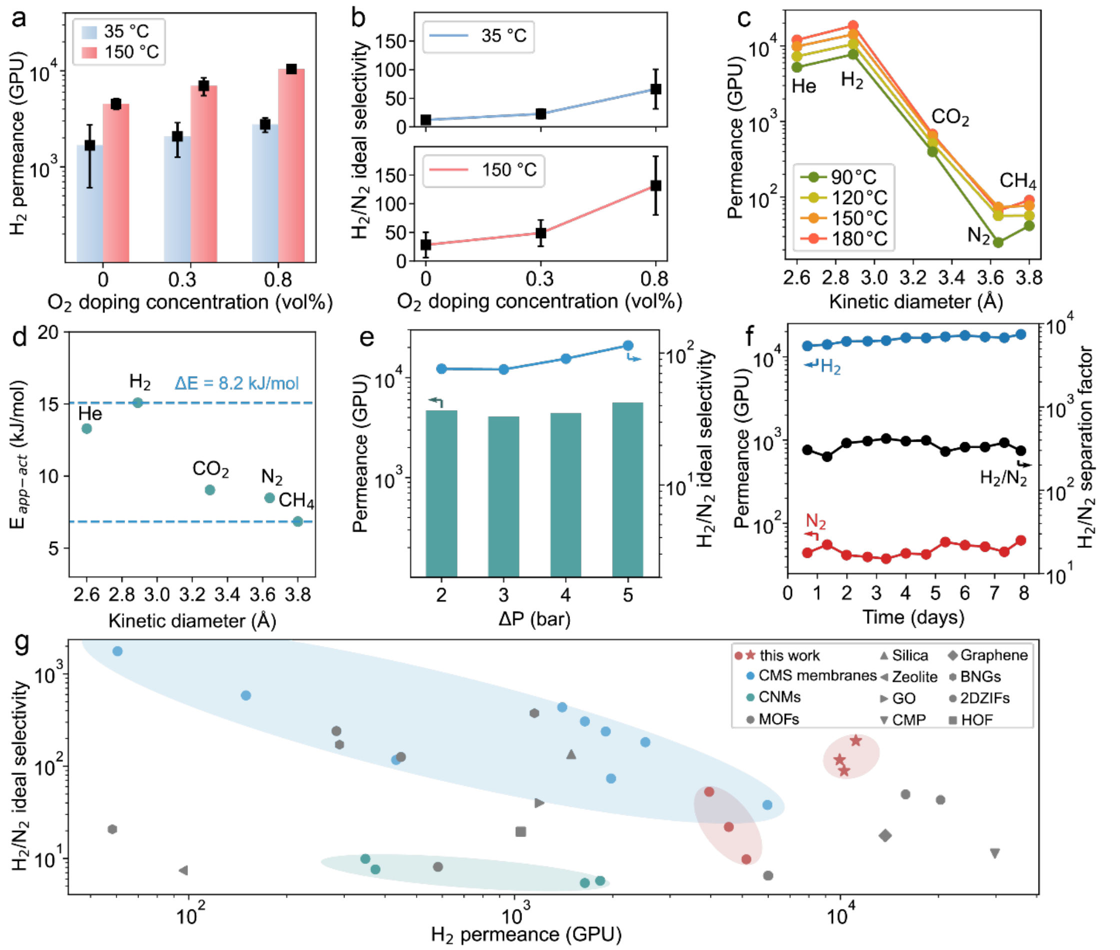

Fig 3. Mechanistic evaluation and benchmarking of UCMs based on gas permeation performance. The influence on the H_2 permeance (**a**) and H_2/N_2 ideal selectivity…(**g**) Comparison of H_2/N_2 ideal selectivity and H_2 permeance (in GPU) among the state-of-the-art CMS membranes for H_2/N_2 separation[9,18,31–35] (blue circles), CNMs[23] (green circles), metal-organic frameworks[36–39] (MOFs, grey circles), silica[40] (grey upward triangle), zeolite[41] (grey leftward triangle), graphene oxide[42] (GO, grey rightward triangle), conjugated microporous polymers[43] (CMP, grey downward triangle), graphene[44] (grey diamond), boron nitride and graphene nanosheets[45] (BNGs, grey hexagons), 2-dimensional zeolitic imidazolate frameworks[46] (2DZIFs, grey octagons), hydrogen-bonded organic framework[47] (HOF, grey square), and this work (d-UCMs, red circles for data at 35°C and red stars, data at 150 °C).

Q 1-7. The apparent activation energy symbol is inconsistent (Eapp−act vs. Eact−app). The legend colors are mismatched in Figure 3 – “blue” should replace “grey” for CMS membranes, and “green” should replace “blue” for CNMs. Also, the color “red” should be corrected to “green” in Figure 6. In supplementary information, Figure S7 contains identical labels C–C sp3 for both orange and green colors. The authors should thoroughly review the figure captions.

Authors' response: We thank the reviewer for the careful check and apologize for these errors in our figures and symbols. We have gone through the entire manuscript and Supporting Information to fix these issues as suggested:

Unified Symbols: We have unified the symbol for apparent activation energy as $E_{app-act}$ throughout the text and figures.

Color Mismatch in Figure 3: We have updated the legend colors. CMS membranes are now shown in blue and CNMs in green.

Correction in Figure 6: The color label has been corrected from "red" to "green". Labels in Figure S7: In the Supporting Information, we have corrected the duplicated C-C sp^3 labels in Figure S7 to properly identify the chemical environments.

Review of Captions: We have also re-checked all other figure captions and legends to make sure they are consistent and accurate.

Revised text and figures:
(Figure 3 and its caption)

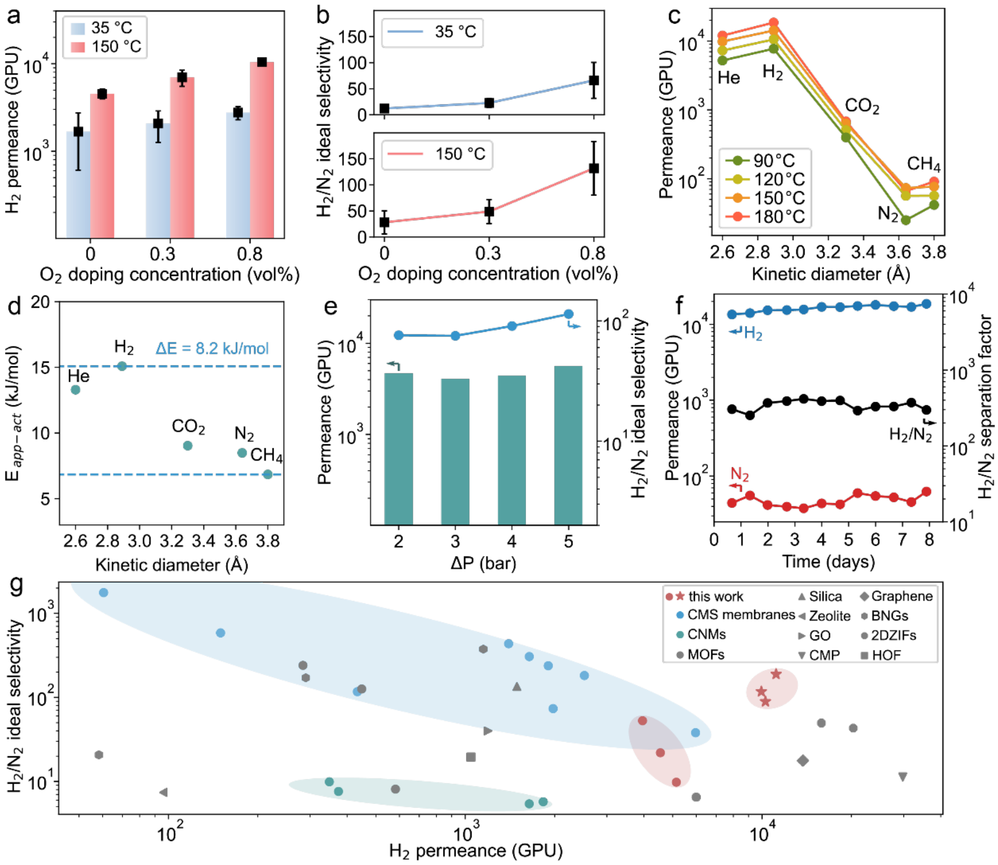

Fig 3. Mechanistic evaluation and benchmarking of UCMs based on gas permeation performance. … (**d**) Apparent activation energy ($E_{app\text{-}act}$) for gas permeation as a function of gas kinetic diameter. ΔE denotes the maximum difference in apparent activation energies between H_2 and CH_4. … (**g**) Comparison of H_2/N_2 ideal selectivity and H_2 permeance (in GPU) among the state-of-the-art CMS membranes for H_2/N_2 separation[9,16,40–44] (blue circles), CNMs[30] (green circles), metal-organic frameworks[45–48] (MOFs, grey circles), silica[49] (grey upward triangle), zeolite[50] (grey leftward triangle), graphene oxide[51] (GO, grey rightward triangle), conjugated microporous polymers[52] (CMP, grey downward triangle), graphene[53] (grey diamond), boron nitride and graphene nanosheets[54] (BNGs, grey hexagons), 2-dimensional zeolitic imidazolate frameworks[55] (2DZIFs, grey octagons), hydrogen-bonded organic framework[56] (HOF, grey square), and this work (d-UCMs, red circles for data at 35°C and red stars, data at 150 °C).

(Figure 6 Caption)

Fig 6. Pyrolysis behavior and decomposition pathways of P4VP in oxidative

and oxygen-free atmospheres. …(**d**) Evolution of MS signal intensity as a function of temperature for major decomposition products, including pyridine (m/z = 73–79), methylpyridine (m/z = 86–88), vinylpyridine (m/z = 104–106), and propenylpyridine (m/z = 117–119) under different pyrolysis conditions (w/o O in black, w O_2 in green).

(Old Figure S7, now Figure S9)

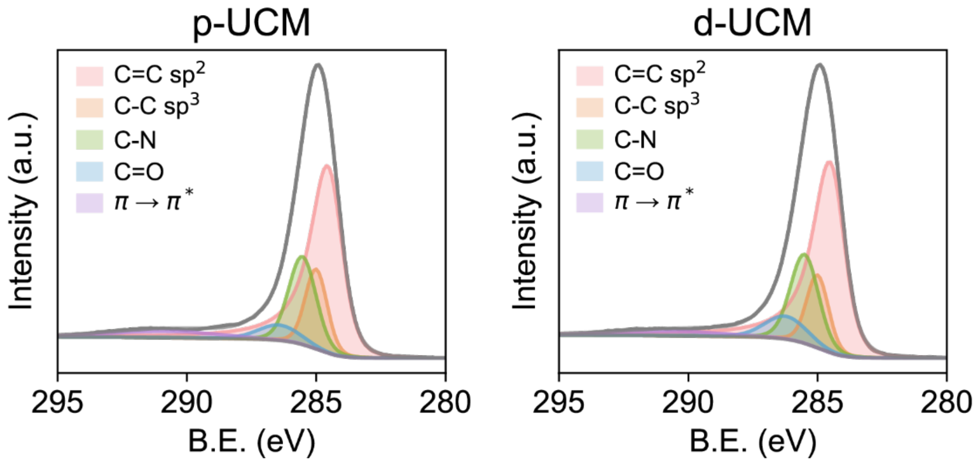

Fig. S9. Peak deconvolution of the initial XPS scan showing C(1s) core level of p-UCM and d-UCM film.

Q 1-8. RRMS values of 0.9 and 1.2 are not identical, though they are close. The use of the term "identical" is overly definitive and should be replaced with a more measured description, such as "similar" or "comparable."

Authors' response: We agree with the reviewer that "identical" is too strong here. We have replaced "identical" with "similar" (or "comparable") in the revised manuscript to be more accurate.

Revised text:
(Discussion, Section 3, Paragraph 2) The root mean square (RMS) roughness measured over a 0.78 μm × 0.78 μm area was similar for p-UCM (0.9 nm) and d-UCM (1.2 nm).

Q 1-9. The interpretation of the GIWAXS results appears somewhat overstated. The observed reflections at 3.4, 3.9, and 5.5 Å are described as evidence of a "hierarchical pore structure" responsible for selective H2 transport. However, GIWAXS primarily captures average interlayer or inter-domain spacings and does not directly resolve the continuous transport pathways or effective pore sizes through which gas molecules diffuse. Consequently, these diffraction features should be discussed as structural motifs correlated with, rather than directly representing, the transport-active free volume.

Authors' response: We thank the reviewer for this insightful comment. We agree that GIWAXS primarily reflects the average inter-domain spacings and doesn't directly represent the transport-active pore pathways.

In the revised manuscript, we have adjusted our discussion to be more cautious. Instead of directly referring the diffraction peaks to "pore size", we now describe the 3.4, 3.9 and 5.5 Å features as structural motifs that represent the structural hierarchy of the d-UCM membranes. We believe that these structural motifs, when correlated

with our gas permeation data, provide a more reasonable interpretation of the structure-performance relationship in our membranes.

Revised text

(Abstract):

We show that O_2 in pyrolysis environment, transforms the otherwise uniform carbon network featuring a ~3.9 Å characteristic interlayer spacing into disrupted UCMs (d-UCMs). These d-UCMs possess a multimodal ultramicroporous structure characterized by distinct d-spacings of ~3.4 Å, 3.9 Å, and 5.5 Å. This optimized distribution of free volume in a 10-nm thick membrane enables a record combination of H_2 permeance exceeding 10,000 gas permeation units (GPUs) and H_2/N_2 mixture selectivity surpassing 200.

(Discussion, Section 3, Paragraph 6)

Tightly packed carbon regions (d-spacing of 3.38 Å) represent compact structural motifs that correlate with enhanced size-sieving transport, consistent with the preferential permeation of H_2 (kd of 2.89 Å) over large N_2 molecules (kd of 3.64 Å). Adjacent broader disordered domains (d-spacing of 5.46 Å) indicate more open structural motifs associated with low-resistance diffusion pathways. This combination of selective sieving and rapid transport allows d-UCMs to achieve significantly enhanced permeance while retaining exceptional selectivity.

(Discussion, Section 3, Paragraph 8)

During p-UCMs synthesis, the dominant intermediates are vinylpyridine (m/z = 105) and pyridine (m/z = 79), accounting for uniform structural motif (~3.9 Å) observed in p-UCMs, a feature that is also displayed in d-UCMs due to their shared intermediates. This interpretation is further supported by prior studies on π–π stacking distances of pyridine (~3.6 Å) and vinylpyridine (~4.2 Å) dimers[58,59], suggesting that the 3.9 Å characteristic spacing arises from stacking interactions between these species. In contrast, TGA-MS analysis of d-UCMs exhibited an abrupt CO_2 release at ~200 °C and an elevated weight loss rate (6 and 9% for p-UCM and d-UCM, respectively, **Fig. 6b**), consistent with the higher nitrogen content due to carbon loss detected by XPS. These results suggest that oxidative pyrolysis preferentially removes less-stable carbon fragments, promoting denser carbon stacking (3.4 Å), which is close to the interlayer spacing of graphitic carbon. Meanwhile, the accumulation of larger pyrolysis intermediates in d-UCMs, such as propenylpyridine (m/z = 119) and methylpyridine (m/z = 93) with extended side chains, disrupts π–π stacking during carbonization[60], leading to the formation of expanded interlayer spacing beyond 3.9 Å in the UCMs.

Reviewer 2

This work reported a so-called ultrathin carbon membrane for H2/N2 separation. the topic is interesting, with a very high H2 permeance was reported. The CVD method was introduced to deposite a unltrathin carbon films for testing. The membrane preparation process is different from the literature reported method for the fabrication of carbon membranes. However, it is different to say this can be a real carbon membrane. And the films cannot be used standalone, and very low potential for real applications. The following issues should be addressed to approve the advances of this work.

Authors' response: We thank the reviewer for these helpful comments. We have addressed the comments and concerns of the reviewer below.

Q 2-1. Hierarchical porosity usually refers to a material with multimodal pore PSD (ultramicropore, micropores), however, all the pores reported in this work ranging 3.38-5.5 Å belong to ultramicropores. It is confusing to call hierarchical porosity. I doubt the pristine carbon membranes have a unique pore size of 3.9Å. based on the relatively low carbonization temperature of 500 ℃, it is highly expected that the prepared carbon membranes are amorphous, which means they may have existing micropores. Gas sorption measurement should be included instead of only with WAXS.

Authors' response: We thank the reviewer for these helpful comments. We agree that "hierarchical porosity" usually implies pores across different scales. Since our detected spacings (3.4–5.5 Å) are all within the ultramicropore range, we have replaced "hierarchical" with "multimodal ultramicroporous structure" throughout the revised manuscript to be more accurate.

Revised text:
(Title)
Oxygen-induced multimodal ultramicroporous structure in 10-nm-thick carbon membranes for high-performance hydrogen separation

(Abstract)
These d-UCMs possess a multimodal ultramicroporous structure characterized by distinct d-spacings of ~3.4 Å, 3.9 Å, and 5.5 Å. This optimized distribution of free volume in a 10-nm thick membrane enables a record combination of H_2 permeance exceeding 10,000 gas permeation units (GPUs) and H_2/N_2 mixture selectivity surpassing 200…Mechanistic investigations reveal that O_2 pyrolysis environment selectively removes relatively weakly-bound carbon species, altering pyrolysis intermediates, resulting in a nitrogen-rich framework with disordered nanodomains and heterogeneous ultramicroporosity.

(Introduction, Paragraph 4)
This unique mechanism created disordered nanodomains and heterogeneous ultramicroporosity within the ultrathin carbon film, enabling ultrafast and highly selective gas separation.

(Discussion, Section 3, title)
Oxygen-induced multimodal ultramicroporous structure in ultrathin carbon membranes

(Discussion, Section 3, Figure 5 caption)
Fig 5. **Structural and chemical modifications in UCM films induced by oxidative**

dosing. (**a**) Conceptual illustration of the structural evolution in UCM films where oxidative pyrolysis leads to a disrupted and more disordered carbon framework (as indicated by d-spacing shifts) and thus changing the gas permeation behavior.

(Discussion, Section 3, Paragraph 5)
This broad distribution of interlayer spacings indicates increased structural heterogeneity, which further supports the role of O_2 in disrupting the carbon network and promoting the formation of a multimodal ultramicroporous structure.

(Discussion, Section 3, Paragraph 6)
This multimodal ultramicroporous structure of d-UCMs explains the observed high H_2/N_2 separation performance. Unlike O-doping strategies typically used for CMS membranes, where pore networks become narrow and where permeance is sacrificed for selectivity, d-UCM synthesis in the oxygen environment leads to a multimodal ultramicroporous structure, improving both permeance and selectivity. Tightly packed carbon regions (d-spacing of 3.38 Å) enable molecular sieving via size sieving, selectively permeating H_2 (kd of 2.89 Å) while rejecting larger N_2 molecules (kd of 3.64 Å) to achieve exceptional H_2/N_2 selectivity.

(Discussion, Section 3, Paragraph 9)
This leads to a more disordered carbon framework with multimodal ultramicroporous structures, in contrast with the more uniform structure of p-UCMs.

(Conclusion)
Controlled oxygen exposure during pyrolysis selectively removes labile carbon bonds while preserving nitrogen-rich domains, resulting in disordered nanostructures with multimodal ultramicroporous networks.

Regarding the membranes in this work, they are indeed amorphous. The 3.9 Å spacing we reported represents the most pronounced inter-domain distance captured by GIWAXS, rather than a single, perfectly uniform pore size. The key point of the manuscript in this respect is that the O_2 dosing significantly disrupted the membrane nanostructure by introducing new, distinct structural motifs. To avoid potential misunderstanding, we have revised the wording in the manuscript accordingly as follows:

Revised text:
(Discussion, Section 3, Paragraph 8) During p-UCMs synthesis, the dominant intermediates are vinylpyridine (m/z = 105) and pyridine (m/z = 79), accounting for predominant structural motif (~3.9 Å) observed in p-UCMs, a feature that is also displayed in d-UCMs due to their shared intermediates.

We agree that gas sorption measurements provide valuable information about the pore structure. We have indeed considered using traditional volumetric adsorption tests; however, we encountered significant practical challenges. Since our selective carbon layer is an ultrathin film (10 nm) with a mass density only on μg/cm^2 level, it is practically impossible to collect a sufficient amount of material for standard bulk-scale test. To address this, we tried our best to design and use a Quartz Crystal Microbalance (QCM) setup (**Figure R9**) to provide reliable CO_2 adsorption data on these thin films. We hope the reviewer understands that this choice was made as the most feasible alternative to capture the intrinsic properties of our membranes given these physical constrains.

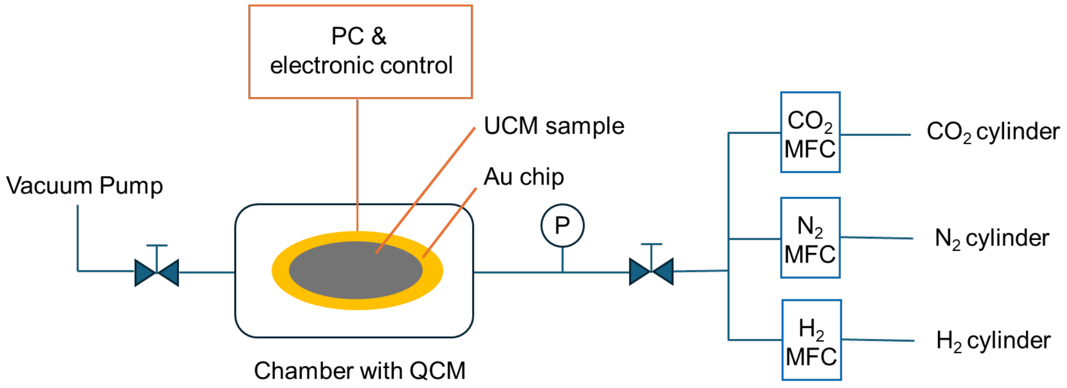

Figure R9. Schematic of the QCM adsorption setup. The ultrathin carbon membrane (UCM) samples are transferred onto a calibrated commercial gold chip. Mass changes during gas adsorption (CO_2, N_2, and H_2) are monitored by tracking the resonance frequency shifts under controlled vacuum and flow conditions. The detection sensitivity is ~ 0.02 µg.

We performed the CO_2 adsorption test and we employed the Horvath-Kawazoe (HK) model to estimate the pore size distribution (the detailed algorithm and equations are provided in the Appendix at the end of the document). As shown in **Figure R10-A**, the CO_2 loading at maximum P/P_0 is approximately 0.4 mmol/g, which is comparable to values reported for other high-performance carbon materials[3].

Based on the loading curves, we observed distinct trends in the PSD for p-UCM and d-UCM (**Figure R10-B**). For the p-UCM (O-free sample), the distribution is relatively narrow with two primary features around 0.7 nm and 0.9 nm. In contrast, the d-UCM (O-dosed sample) exhibits a much broader peak ranging from 0.6 to 1 nm.

We would like to note that the interaction parameter (K) is a critical factor in the HK model, as it describes the interaction between the adsorbent and the adsorbate. Our current calculation assumes a pure graphite-like carbon framework (with parameters listed in the Appendix). However, the presence of nitrogen and oxygen (as confirmed by XPS) likely causes the real K value to deviate from the theoretical one, leading to a shift in the absolute pore size values. Despite this, the broader peak in the d-UCM suggests that the O_2 dosing disrupts the local packing and introduces a more complex pore structure.

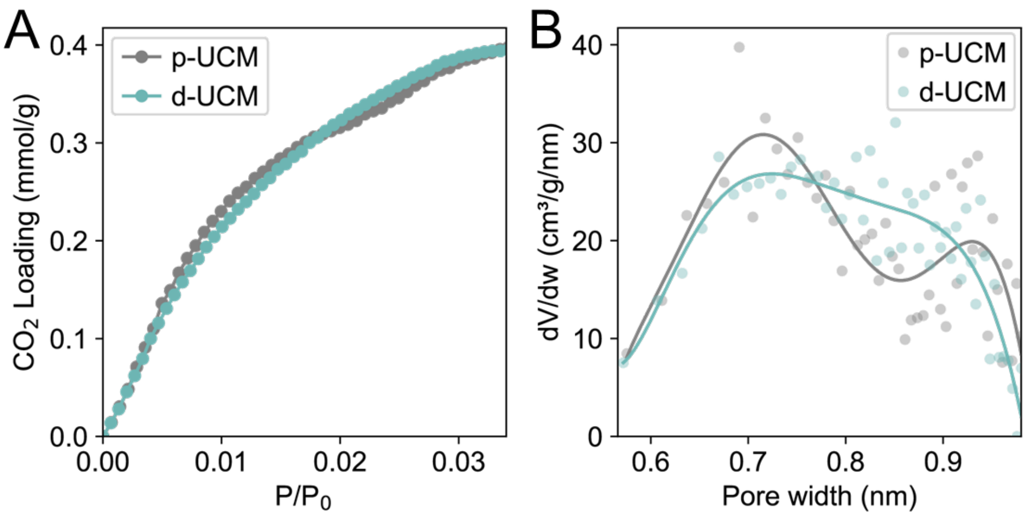

Figure R10. **A**. CO_2 adsorption isotherms at 293 K measured via QCM for the p-UCM

(O-free) and d-UCM (O-dosed) membranes. **B**. Pore size distribution (PSD) curves derived from the HK model. The symbols (dots) represent the discrete PSD results calculated from the adsorption data, while the solid lines are trendlines provided to highlight the structural shift. Data for d-UCM and p-UCM are shown in green and grey, respectively.

Q 2-2. The membrane preparation process is way too complicated compared to other types of carbon membranes reported in the literature. The high gas permeance is dependent on the ultrathin carbon layer of 10nm. However, this makes the upscaling extremely challenging (how the authors expect to scale up the CVD process). From fig. 2A, the prepared carbon membranes have an area of 2 cm2 (actually, not the whole membrane can be used for testing). The authors need to clarify the actual membrane area for gas permeation testing. One critical issue is, compared to supported carbon membranes and hollow fiber carbon membranes (a thorough literature review should be included). How would this membrane be scaled up for future industrial applications? As the authors mentioned, the potential for ammonia cracking process where the gas stream pressure is typically 20-40bar tubular reactor or 50-100bar catalytic reactor. How about the membrane stability at higher pressures (more mixed gas permeation testing should be conducted). the stable performance of these physically stable carbon films (on top of the W support) should be measured. This is a critical issue and should be addressed to document the advances of the developed carbon membranes.

Authors' response: We thank the reviewer for these insightful and practical questions. We have addressed the concerns regarding the literature context, upscaling and the high-pressure stability as follows.

We sincerely appreciate the reviewer's suggestion to include a more comprehensive literature review. We agree that positioning our work within the context of established CMS configurations is essential. Accordingly, we have added a concise but thorough discussion in the Introduction, citing recent representative and pioneering works on supported carbon membranes and hollow fiber membranes. These additions highlight the industrial maturity and high packing density of traditional structures, while providing a clearer benchmark for the permeance breakthrough achieved by our 10 nm ultrathin carbon layer.

Revised text:
(Introduction, Paragraph 2)
Recent advances have brought CMS membrane performance close to industrial separation applications[6], combining excellent selectivity[7–13] with increasing maturity in fabrication[14]. Currently, two primary configurations dominate the CMS landscape: supported thin-film composites on robust porous substrates[15–17] and asymmetric hollow fibers which provide high packing density and a self-supporting structure[18–22]. Both benefit from well-established manufacturing processes originally developed for industrial-scale polymeric and ceramic membranes, providing significant scalability advantages. However, regardless of their geometry, most CMS membranes feature a 3-10 μm thick selective layer to prevent defect formation during the pyrolysis process[4], which inherently limits gas permeance.

The following references have been added:
[15] Jiao, W., Wang, H. J., Zhang, K., & Chung, T. S. (2017). Gas separation performance of supported carbon molecular sieve membranes based on soluble polybenzimidazole. Journal of Membrane Science, 533, 1–10. https://doi.org/10.1016/j.memsci.2017.03.016
[16] Richter, H., Voss, H., Kaltenborn, N., Herzig, S., Wollbrink, A., & Caro, J. (2017). High-Flux Carbon Molecular Sieve Membranes for Gas Separation. Angewandte

Chemie International Edition, 56(27), 7760–7763. https://doi.org/10.1002/anie.201701334
[17] Ma, X., Lin, Y. S., Wei, X., & Kniep, J. (2016). Ultrathin carbon molecular sieve membrane for propylene/propane separation. AIChE Journal, 62(2), 491–499. https://doi.org/10.1002/aic.15018
[18] Ma, C., & Koros, W. J. (2013). Ester-Cross-linkable Composite Hollow Fiber Membranes for CO2 Removal from Natural Gas. Industrial & Engineering Chemistry Research, 52(31), 10495–10505. https://doi.org/10.1021/ie4005697
[19] Qiu, W., Zhang, C., Itta, A. K., Liu, G., & Koros, W. J. (2019). Hyperaging Tuning of a Carbon Molecular-Sieve Hollow Fiber Membrane with Extraordinary Gas-Separation Performance and Stability. Angewandte Chemie International Edition, 58(34), 11700–11703. https://doi.org/10.1002/anie.201905641
[20] Ma, C., Itta, A. K., & Koros, W. J. (2015). Thin-skinned intrinsically defect-free asymmetric mono-esterified hollow fiber precursors for crosslinkable polyimide gas separation membranes. Journal of Membrane Science, 493, 252–262. https://doi.org/10.1016/j.memsci.2015.06.012
[21] Lei, L., Pan, F., Lindbråthen, A., Zhang, X., Hillestad, M., Nie, Y., Bai, L., He, X., & Guiver, M. D. (2021). Carbon hollow fiber membranes for a molecular sieve with precise-cutoff ultramicropores for superior hydrogen separation. Nature Communications, 12(1), 268. https://doi.org/10.1038/s41467-020-20628-9
[22] Lei, L., Pan, F., Lindbråthen, A., Zhang, X., & He, X. (2019). Screening Cellulose Spinning Parameters for Fabrication of Novel Carbon Hollow Fiber Membranes for Gas Separation. Industrial & Engineering Chemistry Research, 58(30), 13330–13339. https://doi.org/10.1021/acs.iecr.9b01931

Regarding the complexity of the CVD-based process and related upscaling, while the current synthesis procedure is at a small laboratory scale, CVD is a well-established technology with commercial production by roll-to-roll synthesis procedure. A very successful example is roll-to-roll production of single-layer graphene which is commercial now. Our group has recently demonstrated the successful upscaling to 50 cm^2 size for porous single-layer graphene membranes using special designed modules[4], which could be also adopted in the future for ultrathin carbon membranes in this work. We have active ongoing project on roll-to-roll production of porous graphene membrane (https://s4s-ccus.epfl.ch), and have a roll-to-roll production tool available in our laboratory.

To clarify the size of the membrane, the permeation data were obtained using a macroporous tungsten (W) substrate hosting 5-μm-sized pore arrays as previously reported and the total membrane area is ~ 1 mm^2. Currently, we are fabricating membranes with membrane area of ~1 cm^2 (shown in **Figure R11**) using a specially designed module that was recently reported by our group to evaluate the potential of these membranes for practical industrial applications. A representative large-area membrane exhibits a H_2 permeance of ~4500 GPU and H_2/N_2 selectivity of ~12. While this represents a first attempt at scaling up, it demonstrates the potential of ultrathin carbon membrane for future development toward industrial applications.

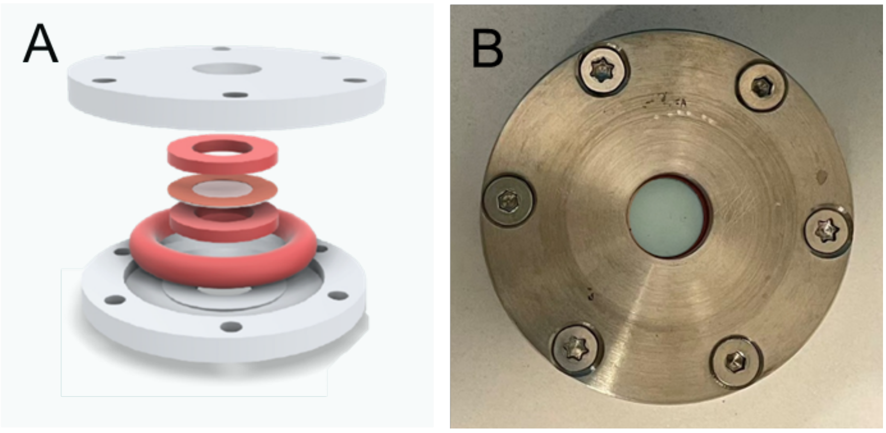

Figure R11. **A**. Schematic illustration of the membrane module architecture designed by our group for upscaling. **B**. Photograph of the UCM membrane fabricated using this method, with the blue region indicating the effective membrane area (~ 1 cm^2).

We agree with the reviewer that high pressure stability is extremely important for the membrane process. For this, we evaluated the mechanical robustness of our membranes through pressure-dependent permeation tests up to the safety limit of our lab-scale setup (6.3 bar transmembrane pressure difference, ΔP). As shown in Table R1, the membrane remained operational with structural integrity, maintaining a stable H_2 permeance of approximately 5.1×10^{-7} mol m^{-2} s^{-1} Pa^{-1}.

Table R1. Single gas permeation results of H_2 and N_2 at varying feed pressures (transmembrane pressures, permeate side at ~ 0 bar) from 2 - 6.3 bar at 35 °C.

**Feed pressure**	**H_2 (mol m^{-2} s^{-1} Pa^{-1})**	**N_2(mol m^{-2} s^{-1} Pa^{-1})**	**Selectivity**
2	5.1×10^{-7}	2.2×10^{-8}	23
3	5.2×10^{-7}	1.5×10^{-8}	36
4	4.8×10^{-7}	1.1×10^{-8}	45
5	5.1×10^{-7}	9.0×10^{-9}	57
6	5.2×10^{-7}	7.6×10^{-9}	69
6.3	5.2×10^{-7}	8.3×10^{-9}	62

While our current setup limits us to ~6 bar, the stable performance at this pressure is a strong proof-of-concept.

Q 2-3. The mixed gas permeation testing shows higher H_2/N_2 selectivity compared to that of single gas permeation testing results. The authors need to elaborate more. Both H_2 and N_2 may have relatively low adsorption capacity (gas sorption experiment should be included).

Authors' response: We thank the reviewer for this insightful comment. We performed additional permeation experiments using single gases and H_2/N_2 gas mixtures with different compositions (80/20, 50/50, and 20/80) on the same membrane at 150 °C. The permeances of H_2 and the H_2/N_2 selectivity are shown in **Figure R8**.

As shown in **Figure R8**, the H_2 permeance (~4.9×10^{-6} mol m^{-2} s^{-1} Pa^{-1}) and gas pair selectivities in all binary gas tests remains nearly identical for single gas and gas mixtures, with a slight improvement in selectivity for gas mixture. This demonstrates that competitive transport effects are weak. This is mainly because the transport of

hydrogen is in the molecular sieving regime, and hydrogen and nitrogen do not have strong binding affinities in porous carbon.

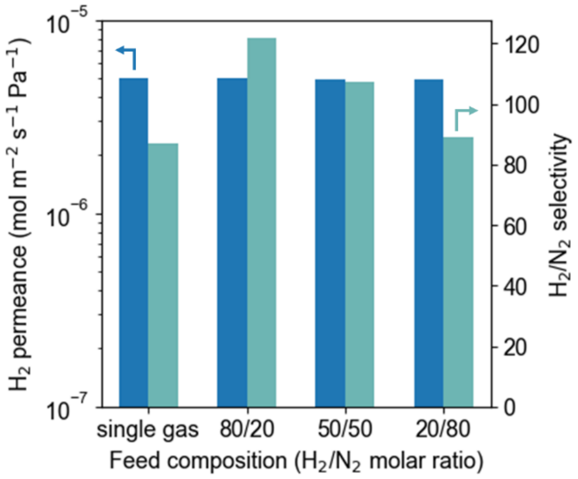

Figure R8. Gas permeation test of d-UCM membrane at 150°C with binary feed gas.

Regarding the gas adsorption test, we have to emphasize that traditional volumetric adsorption tests are not practical given our membrane thickness is only 10 nm with a mass density only on the μg/cm^2 level. Given these extreme constrains, the total gas uptake falls below the sensitivity threshold of standard equipments. To achieve required sensitivity, we designed and used a highly sensitive QCM setup to test the adsorption of N_2 and H_2. As shown in **Figure R12**, the N_2 adsorption capacity of d-UCM samples is ~0.25 mmol/g at 2 bar, which aligns well with experimental and simulation values reported recently for typical carbon molecular sieve materials[5,6]. This validates the accuracy of our measurements even on the ultrathin film. For H_2, the adsorption was below the detection limit of the setup, which is expected due to its small molecular weight (2 g/mol) and weak adsorption behavior.

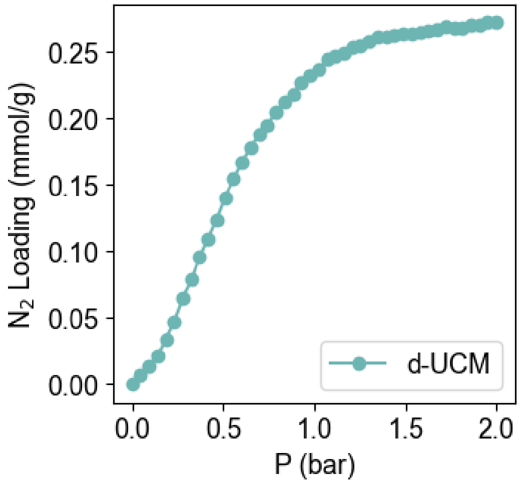

Figure R12. N_2 adsorption curve at 293 K for d-UCM (O-dosed sample) measured by QCM setup.

We have revised the manuscript to include this important point raised by the reviewer as follows:

Revised text:
(Discussion, Section 2, Paragraph 2)
The 0.8 vol% case led to the best performance. For example, a membrane exhibited

an ideal selectivity of 189 with a H_2 permeance of 11145 GPU at 150°C. To evaluate the separation performance under industrially relevant conditions, binary mixed-gas tests were conducted with varied feed compositions (**Fig. S7**). The resulting mixed-gas permeance and H_2/N_2 selectivity remained comparable to single-gas results, which suggests that competitive transport and sorption effects are negligible in the d-UCM membranes. This is consistent with adsorption data obtained via a quartz crystal microbalance (QCM) for the d-UCM film (**Fig. S8**). The N_2 adsorption capacity was ~0.25 mmol/g at 2 bar, while the H_2 adsorption was below the detection limit. These results suggest the weak binding affinity between the gas molecules and the carbon framework. Notably, all membranes exhibited higher H_2 permeance and higher H_2/N_2 selectivity at elevated temperatures, indicating a temperature-activated H_2 transport.

(Methods)

1.5.8. Gas adsorption measurements through Quartz Crystal Microbalance

Gas adsorption of the ultrathin d-UCM film was measured using a Quartz Crystal Microbalance with Dissipation monitoring (QCM-D, open QCM, Italy) setup capable of recording multiple overtones simultaneously inside a vacuum chamber (**Fig. S13**). The UCM sample was transferred onto a 5 MHz gold-coated quartz sensor using the same method as used for the membrane preparation. After drying at 100 °C and evacuating the system to achieve a stable baseline, the chamber was gradually pressurized with the target gas (H_2 or N_2) up to 2 bar. The mass change (Δm) due to gas adsorption was calculated from the frequency shift (Δf) using the Sauerbrey equation:

$$\Delta m = -\frac{C\Delta f}{n}$$

where C is the mass sensitivity constant and n is the overtone number. A control experiment with a bare gold sensor showed negligible frequency change, confirming that the observed signals originated from adsorption on the UCM films.

Added figures:

Figure R8, Figure R9 and Figure R12 are added as Fig. S7, Figure S8 and Fig. S13 to the supplementary information.

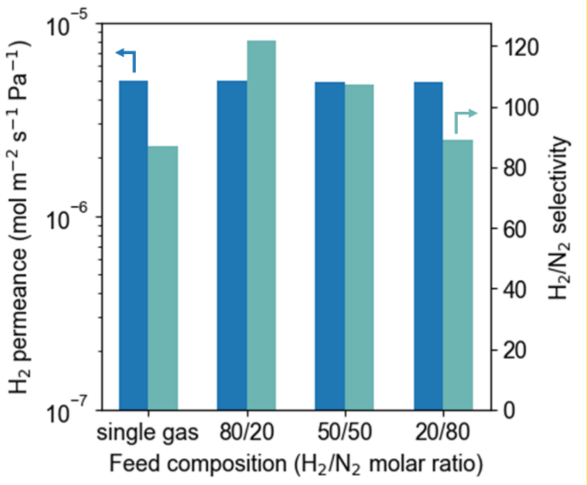

Fig. S7 Gas permeation performance of the d-UCM membrane at 150°C under single feed gas and binary feed gas (H_2/N_2 ratios: 80/20, 50/50, 20/80) conditions.

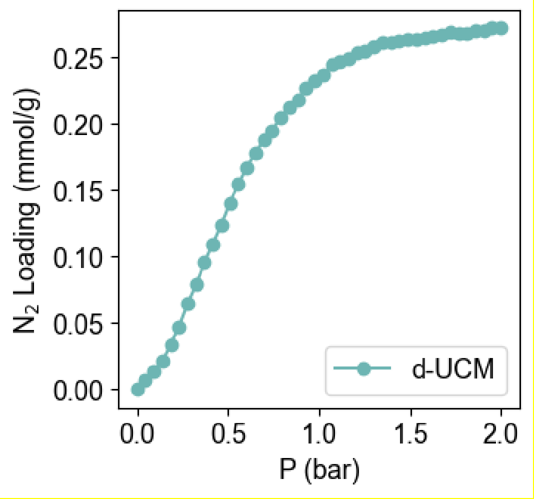

Fig. S8. N_2 adsorption curve at 293 K for d-UCM (O-dosed sample) measured by QCM setup.

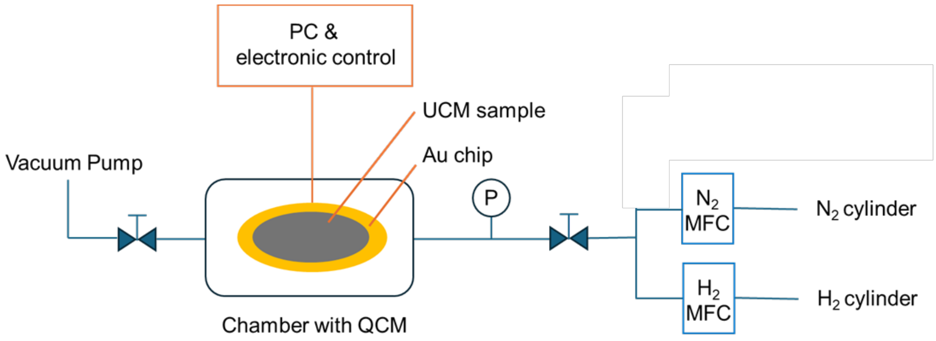

Fig. S13 Schematic of the QCM adsorption setup. The ultrathin carbon membrane (UCM) samples are transferred onto a calibrated commercial gold chip. Mass changes during gas adsorption (N_2, and H_2) are monitored by tracking the resonance frequency shifts under controlled vacuum and flow conditions. The detection sensitivity is ~ 0.02 μg.

Q 2-4. Looking into the hydrogen economy, H_2 production from SMR and biohydrogen, as well as H_2 recovery from industrial waste gas streams is of great interest. Why the authors believe H_2/N_2 separation is an urgent issue, which is less challenging compared to H_2/CO_2 separation. How about the competitiveness of H_2/CO_2 separation for the developed carbon membranes.

Authors' response: We appreciate the reviewer's perspective. While H_2/CO_2 is an important separation pair, we chose to focus on H_2/N_2 primarily due to its increasing importance in the ammonia economy, while efficient H_2/N_2 separation is a critical step there.

Regarding H_2/CO_2 separation, our membranes exhibit a selectivity of ~ 23 with H_2 permeance >10000 GPU at 150 °C. These results confirm that the ultrahigh H_2 permeance, enabled by our oxygen-dosing strategy and the ultrathin membrane structure, is promising for molecular sieving across various H_2-relevant gas pairs.

Q 2-5. For mechanical strength, the authors stated that "The lower Young's modulus of the d-UCM is comparable to that of polybenzimidazole (PBI), making UCMs suitable for integration into a practical membrane". A comparison with the literature reported

carbon membranes (e.g., PI or cellulose derived (>7.5GPa)) should be included. Instead of comparing with a polymeric membrane.

Authors' response: We thank the reviewer for this constructive suggestion. Our initial comparison with PBI was intended to demonstrate that the d-UCM possesses sufficient mechanical integrity to serve as a functional membrane, as it meets the mechanical thresholds typically required for high-performance polymeric separators. We have followed the advice and also included a comparison with literature-reported carbon membranes derived from cellulose, copolyimide and polybenzimiazole as shown in the revised manuscript (see **Figure 2c**).

Revised figure and its caption:

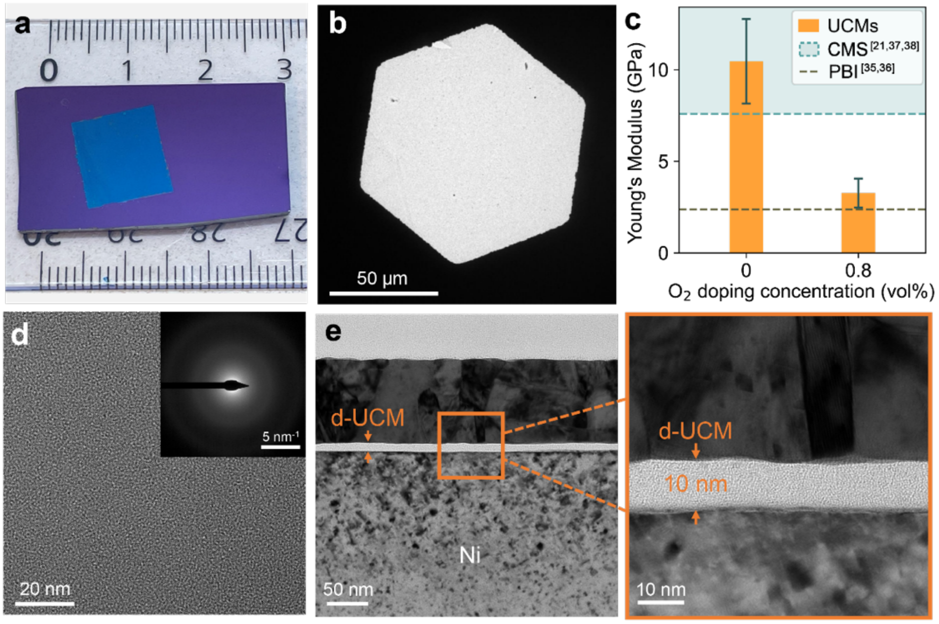

Fig. 2. Structural characterization of UCMs. (a) Optical image of a 1 cm × 1 cm d-UCM film transferred onto a Si wafer. (b) TEM image of a free-standing d-UCM on a hexagonal copper grid. (c) Elastic modulus of p-UCM (0 vol% O_2) and d-UCM (0.8 vol% O_2), compared with hydrogen-selective polymeric film (polybenzimidazole (PBI)) and carbon molecular sieve membranes reported in the literature[21,35–38]. (d) Top-view HRTEM image of the free-standing d-UCM and the corresponding diffraction pattern of the same area. (e) Cross-sectional HRTEM image of the d-UCM on a Ni substrate from an FIB-prepared lamella and the corresponding zoom-in cross-sectional HRTEM image showing the d-UCM thickness of 10 nm and its amorphous carbon structure.

The following references have been added:

[21] Lei, L., Pan, F., Lindbråthen, A., Zhang, X., Hillestad, M., Nie, Y., Bai, L., He, X., & Guiver, M. D. (2021). Carbon hollow fiber membranes for a molecular sieve with precise-cutoff ultramicropores for superior hydrogen separation. *Nature Communications*, *12*(1), 268. https://doi.org/10.1038/s41467-020-20628-9

[37] Hu, L., Bui, V. T., Krishnamurthy, A., Fan, S., Guo, W., Pal, S., Chen, X., Zhang, G., Ding, Y., Singh, R. P., Lupion, M., & Lin, H. (2022). Tailoring sub-3.3 Å ultramicropores in advanced carbon molecular sieve membranes for blue hydrogen production. *Science Advances*, *8*(10), eabl8160. https://doi.org/10.1126/sciadv.abl8160

[38] Wang, K., Zhu, Z., Liu, Y., Zheng, W., Yuan, Z., Lin, Z., Semiat, R., Shao, L., & He, X. (2025). Adsorption-enhanced carbon membranes derived from copolyimide for ultrafast subangstrom discriminating CO 2 separation. *Science Advances*, *11*(48),

eadv8650. https://doi.org/10.1126/sciadv.adv8650

Q 2-6. The d-spacing (d(002) detected by WAXS) reflects the distance between carbon planes, not exactly the pores as illustrated in Fig. 5A. The larger d-spacing (compared to Graphite) indicates the amorphous carbon structure. The authors use d-spacing to illustrate that the pore channel of the developed carbon membranes may lead to a misunderstanding for the readers; this must be clarified. Gas sorption measurements using H2 or CO2 should be conducted to characterize the PSD (instead of giving an illustration in Fig. 5a).

Authors' response: We thank the reviewer for the constructive comments which helped to improve the quality of the manuscript. We agree that the d-spacing obtained from WAXS reflects the average distance between disordered carbon layers, which, while indicative of the overall framework density, should not be directly equated with the kinetic pore diameter for gas transport. The original illustration in **Figure 5A** was intended purely as a conceptual schematic to visualize the framework expansion; to avoid any reader misunderstanding, we have revised the figure caption to clarify its illustrative nature.

Revised figure caption:
Fig 5. **Structural and chemical modifications in UCM films induced by oxidative dosing.** (**a**) Conceptual illustration of the structural evolution in UCM films where oxidative pyrolysis leads to a disrupted and more disordered carbon framework (as indicated by d-spacing shifts) and thus changing the gas permeation behavior.

Regarding gas sorption and PSD characterization, as detailed in our response to **Question 2-1** (please refer to **Figure R9** and **R10**), we have performed CO_2 gas adsorption measurements to evaluate the pore size distribution (PSD). The PSD curves derived from the Horvath-Kawazoe (HK) model provide definitive evidence of the pore structural evolution. These experimental results confirm that O_2 dosing disrupted the relatively uniform ultramicropores of p-UCM into a broader, multimodal distribution in d-UCM.

Q 2-7. The prepared film is formed by the deposition of the released small molecules such as propenylpyridine (m/z =119), methylpyridine (m/z = 93), and pyridine and vinylpyridine based on CVD. It's totally different from the traditional carbon membranes based on the formed carbon plates (mechanically stronger), which means the form films cannot be used standalone, and must be aligned to a support. it might be only for research purposes.

Authors' response: We thank the reviewer for this valuable perspective. We would like to clarify this point from two aspects.

Regarding the different synthesis strategy, we agree that our method utilizes the pyrolysis vapor as the precursor for carbon film growth, which is different from the traditional carbon molecular sieve fabrication that focuses on the solid residue of pyrolysis. By dosing oxygen into the growth atmosphere, we achieved a simultaneous improvement in both selectivity and permeance. While this work is currently fundamental and research-oriented, we hope this novel approach of vapor-phase engineering strategy can foster the development of ultrathin carbon material for high permeance gas separation applications.

Regarding the requirement for a support, we believe that this is a common strategy

within the whole membrane community to overcome the trade-off between thickness and flux. Several commercialized high-performance membranes for gas separation are based on thin-film composite architecture where a porous support, gutter layer, selective layer, and pore masking layer are used in combination. These kinds of asymmetric membranes are highly desired as they allow the dense selective layer to provide precise molecular sieving while the porous support layer provides sufficient mechanical reinforcement for practical operation. In our research, by decoupling the selective layer from the reinforcing layer, we were able to reduce the selective layer to just 10 nm. This combination achieves ultra-high gas permeance that significantly improves overall membrane efficiency.

Appendix

The original Horvath-Kawazoe Model was designed to calculate the slit-like channels in carbon molecular sieving materials with pore diameters in the order of 0.1 nm. The model assumes that the C material channel is based on a slit-shaped pore with two parallel layers of graphene domains. The original Horvath-Kawazoe Method uses this following equation to calculate the pore size:

$$\Phi = \frac{N_a A_a + N_A A_A}{2\sigma^4}\left[-\left(\frac{\sigma}{r}\right)^4 + \left(\frac{\sigma}{r}\right)^{10} - \left(\frac{\sigma}{1-r}\right)^4 + \left(\frac{\sigma}{1-r}\right)^{10}\right]$$

Where N_a is the number of atoms per unit area of surface; A_a is a constant given by the Kirkwood-Muller, London, etc., equations. σ is the distance between a gas atom and the surface at zero interaction energy. r is the distance from the surface. N_a is the number of atoms per unit area of surface, A_a is a constant given by the Kirkwood-Muller, London, etc., equations. N_A is the number of molecules per unit area of the adsorbate and A_A is another constant in Lennard-Jones potential. A_A and A_a can be calculated with the following equation:

$$A_A = \frac{3mc^2 \alpha_A \chi_A}{2}$$

$$A_a = \frac{6mc^2 \alpha_a \alpha_A}{\frac{\alpha_a}{\chi_a} + \frac{\alpha_A}{\chi_A}}$$

The parameters used here are:

	Symbol (units)	Adsorbent: Carbon (Graphite)	Adsorbate: CO_2
Electron mass	m (kg)	9.11×10^{-31}	
Light speed	c (cm/s)	3×10^{10}	
Susceptibility	χ (10^{-29} cm^3)	13.5	3.50
Polarizability	α (10^{-24} cm^3)	1.02	2.63
Surface atomic density	N ($atoms/cm^2$)	3.845×10^{15}	1.03×10^{15}

This equation can be transformed into the following equation[8]:

$$ln(P/P_0) = \frac{K_{interaction}}{(l-d)} \times \left[\frac{\sigma^4}{3\left(l-\frac{d}{2}\right)^3} - \frac{\sigma^{10}}{9\left(l-\frac{d}{2}\right)^9} - \frac{\sigma^4}{3\left(\frac{d}{2}\right)^3} + \frac{\sigma^{10}}{9\left(\frac{d}{2}\right)^9}\right]$$

Where:

$$K_{interaction} = \frac{N_A A_A + N_a A_a}{RT\sigma^4}$$

$$d = d_C + d_{CO_2}$$

$$\sigma = 0.858\frac{d}{2} = 0.858\frac{d_C + d_{CO_2}}{2}$$

The numbers used in this equation are listed in the following table:

d_C [nm]	0.34	σ [nm]	0.266
d_{CO_2} [nm]	0.28	$K\_interaction$	39.56
d [nm]	0.62		

Then the equation becomes:

$$\ln\left(\frac{P}{P_0}\right) = \frac{39.56}{l - 0.64} \times \left[\frac{1.669 \times 10^{-3}}{(l - 0.31)^3} - \frac{1.970 \times 10^{-7}}{(l - 0.31)^9} - 0.0486\right]$$

By fitting the l with $\ln(P/P_0)$, we can get that for each P/P_0 condition, pores of size l will be filled.

References

1. Lee, C.-K. *et al.* Monatomic Chemical-Vapor-Deposited Graphene Membranes Bridge a Half-Millimeter-Scale Gap. *ACS Nano* **8**, 2336–2344 (2014).

2. Huang, S. *et al.* Single-layer graphene membranes by crack-free transfer for gas mixture separation. *Nat. Commun.* **9**, 2632 (2018).

3. Iyer, G. M., Ku, C.-E. & Zhang, C. Hyperselective carbon membranes for precise high-temperature H2 and CO2 separation. *SclEnce AdvAnceS* (2025).

4. Hao, J. *et al.* Scalable synthesis of CO2-selective porous single-layer graphene membranes. Preprint at https://doi.org/10.26434/chemrxiv-2025-z8g2d (2025).

5. Hou, M. *et al.* Unraveling the relationship between microstructure of CMS membrane and gas transport property using molecular simulation. *AIChE Journal* **70**, e18561 (2024).

6. Andrade, M., Rodrigues, S. C. & Mendes, A. High performing CMS adsorbent for O2 / N2 separation. *Microporous and Mesoporous Materials* **296**, 109989 (2020).

7. Yin, Y. & Collins, R. E. Carbon molecular sieve films produced by dc sputtering. *Carbon* **31**, 1333–1336 (1993).

8. Horváth, G. & Kawazoe, K. Method for the calculation of effective pore size distribution in molecular sieve carbon. *J. Chem. Eng. Japan / JCEJ* **16**, 470–475 (1983).
